# Methodological differences can affect sequencing depth with a possible impact on the accuracy of genetic diagnosis

**DOI:** 10.1590/1678-4685-GMB-2019-0270

**Published:** 2020-04-27

**Authors:** Murilo G. Borges, Cristiane S. Rocha, Benilton S. Carvalho, Iscia Lopes-Cendes

**Affiliations:** 1 Universidade Estadual de Campinas (UNICAMP), Faculdade de Ciências Médicas, Departamento de Genética Médica e Medicina Genômica, Campinas, SP, Brazil.; 2 Instituto Brasileiro de Neurociência e Neurotecnologia (BRAINN), Campinas, SP, Brazil.; 3 Universidade Estadual de Campinas (UNICAMP), Instituto de Física “Gleb Wataghin”. Campinas, SP, Brazil.; 4 Universidade Estadual de Campinas (UNICAMP), Instituto de Matemática, Estatística e Computação Científica, Departamento de Estatística, Campinas, SP, Brazil.

**Keywords:** Whole exome sequencing, depth, ClinVar, computational biology, clinical genomics

## Abstract

For a better interpretation of variants, evidence-based databases, such as ClinVar, compile data on the presumed relationships between variants and phenotypes. In this study, we aimed to analyze the pattern of sequencing depth in variants from whole-exome sequencing data in the 1000 Genomes project phase 3, focusing on the variants present in the ClinVar database that were predicted to affect protein-coding regions. We demonstrate that the distribution of the sequencing depth varies across different sequencing centers (pair-wise comparison, *p* < 0.001). Most importantly, we found that the distribution pattern of sequencing depth is specific to each facility, making it possible to correctly assign 96.9% of the samples to their sequencing center. Thus, indicating the presence of a systematic bias, related to the methods used in the different facilities, which generates significant variations in breadth and depth in whole-exome sequencing data in clinically relevant regions. Our results show that methodological differences, leading to significant heterogeneity in sequencing depth, may potentially influence the accuracy of genetic diagnosis. Furthermore, our findings highlight how it is still challenging to integrate results from different sequencing centers, which may also have an impact on genomic research.

## Introduction

Whole exome sequencing (WES) has emerged as a powerful tool in genomic medicine as it provides the possibility of interrogating the genome in its most interpretable portion (Coffey *et al.*, 2011; Mahon, 2016). This strategy has identified causal variants in several Mendelian diseases with a high success rate (Gilissen *et al.*, 2012; Shamseldin *et al.*, 2017). Therefore, the use of WES has proven to add relevant diagnostic information, and it is currently widely used in medical practice (Linderman *et al.*, 2014; Suwinski *et al.*, 2019; Ulintz *et al.*, 2019). However, several methodological issues can affect the results obtained by WES and may influence its interpretation (Sulonen *et al.*, 2011; Hardwick *et al.*, 2017).

The capture experiment, followed by the enrichment phase, is a crucial step to ensure success in WES since it is essential to determine reads uniformity, depth, and overall quality of sequencing (Chilamakuri *et al.*, 2014; Wang *et al.*, 2017). One of several parameters used for quality control on massively parallel DNA sequencing experiments is the depth of coverage, which refers to the average number of sequenced and adequately aligned bases or reads to a specific genomic position or region (Elmas *et al.*, 2018). Its expected value is one of the first parameters to be estimated in the study design of a given sequencing experiment (Sims *et al.*, 2014). In WES, the depth varies greatly, so that even when the expected average depth is high, the capture of some regions may still be problematic, leading to an uneven distribution of sequencing depth (Lelieveld *et al.*, 2015). It is well-known that the results obtained from massively parallel DNA sequencing technologies may suffer some biases due to the experimental design, sample selection, sequencing strategies, and variant calling methods (Asan *et al.*, 2011; Hwang *et al.*, 2016; Meienberg *et al.*, 2016; Van Allen *et al.*, 2016). In this context, we aimed to analyze the pattern of sequencing depth in variants from WES data in the 1000 Genomes project phase 3, focusing on the variants present in the ClinVar database that were predicted to affect proteincoding regions.

## Materials and Methods

We used the public binary alignment map files (BAM) available from the 1000 Genomes Project Consortium FTP web page to calculate the depth of sequencing variations from ClinVar entries in 1,112 WES samples from sequencing phase 3 (Table S1). We guarantee the integrity of the analyzed BAM files by automatically generating and checking the MD5 code of each downloaded file by implementing an automatic script. If there were any discrepancies between the MD5 codes provided by the 1000 Genomes Project and the one obtained by us, we performed the download once again. The samples were all sequenced in an Illumina HiSeq 2000 with a paired-end sequencing reaction in four different sequencing facilities listed below. Each center participating in the consortium applied a different WES capture methodology: the Baylor College of Medicine (BCM) applied a customized array HSGC VCRome, the Broad Institute (BI) used Agilent SureSelect All Exon v2, the Beijing Genomics Institute (BGI) used NimbleGenSeqCap EZ Exome v2, and the Washington University Genome Center (WUGC) used NimbleGenSeqCap EZ Exome v3.

We extracted 282,453 variants from ClinVar (built 20170801, GRCh37.p13) (Landrum *et al.*, 2018) and performed variant annotation using the Ensembl Variant Effect Predictor (VEP version 84) using the default parameters (McLaren *et al.*, 2016). Overall, 4,543 variants were classified as exonic in the autosome chromosomes and had a predicted impact on mRNA and protein structure and function (121 were classified as high, 2,166 moderate, 1,641 low, and 615 as a modifier). We provide the variant calling file containing these targets as File S2. We used “samtools depth” (version 1.3.1) to estimate the base-by-base depth of the 4,543 selected variants for each of the BAM files, accepting reads with sequencing and mapping quality greater than 30 (99.9% reliability) (Li *et al.*, 2009; Li, 2011). We then performed the merging of each of the BAM files with the coverage of our ClinVar targets.

We conducted all further analyses using the R statistical environment (version 3.3.2) (R Core Team, 2014). First, we tested the assumption of no difference in the pattern of sequencing depths in each of the four sequencing centers with a Mann-Whitney-Wilcoxon test with continuity correction in the normal approximation for the p-value. We also applied a multidimensional scaling (MDS) method over the resulting depth in each region and compared the different groups, addressing the data high-dimensionality issue, and obtained a low-dimensional representation of the data (Kruskal and Wish, 1978). We show the results obtained using R packages to process and generate conventional and interactive charts (plyr 1.8.4, plotly 4.8.0, ggplot2 3.0.0). Furthermore, we visually recorded the variation in depth of sequencing in the different sequencing centers with a heatmap (heatmaply 0.9.1) of the 450 variants, which presented the higher variance across samples. We apply a method of clustering to this high variability subset of targets by using the k-means algorithm, considering a total of 5 groups (Macqueen, 1967).

## Results

**Figure 1:**
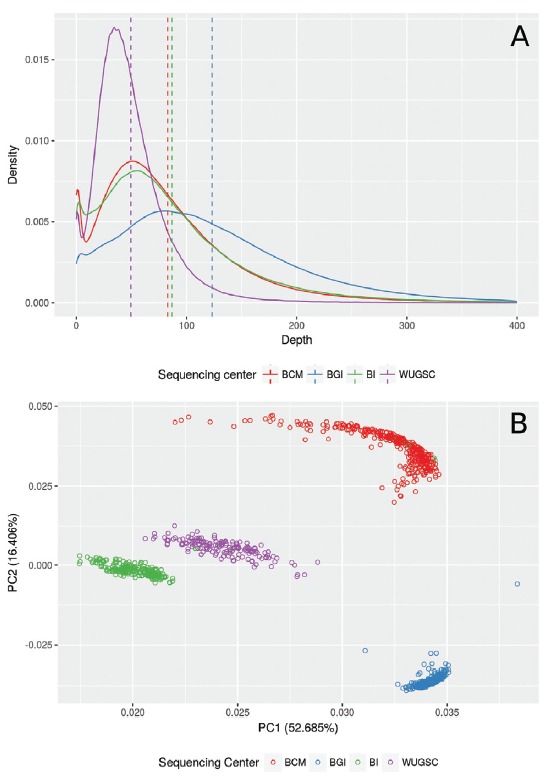
Variation in depth of sequencing in different sequencing centers. Depth distribution varies significantly (p < 0.001) among samples from the four sequencing centers included in this study (BCM Baylor College of Medicine, BI Broad Institute, BGI, and WUGC Washington University Genome Center). (A) Density distributions for regions from ClinVar with depth from 0 to 400, with an average of 82.8 ± 67.6 for BCM, 123.0 ± 85.6 for BGI, 86.6 ± 79.2 for BI, and 49.4 ± 33.8 for WUGSC. (B) Principal component analysis (PCA) corroborates our findings, with an explained variance of 69.0% for the first two components. Complete depth distribution and an interactive 3D version of Figure 1B is available as File S3.

The average sequencing depth from the selected 4,543 variants from ClinVar differed significantly among the sequencing centers (pairwise comparisons with MannWhitney-Wilcoxon test, *p* < 0.001), with an average depth of 82.8 ± 67.6 for the BCM, 123.0 ± 85.6 for the BGI, 86.6 ± 79.2 for the BI, and 49.4 ± 33.8 for the WUGSC (Figure 1A, File S2).

The multidimensional scaling analysis corroborates that the pattern of sequencing depth clusters according to each sequencing center, with 69% of the variance explained by the first two principal components in the principal component analysis (PCA, Figure 1B, File S2). This indicates that protocol advancement and intrinsic methodological differences in each of the sequencing centers directly affect the pattern of the sequencing depth in the set of variants analyzed. The inconsistency in the depth and breadth of coverage across samples introduces a systematic bias in the results generated by each center. Sequencing depth is a measurement of how many times a certain variant was sequenced while the breadth is the capability of adequately capturing and sequencing a given region.

**Figure 2:**
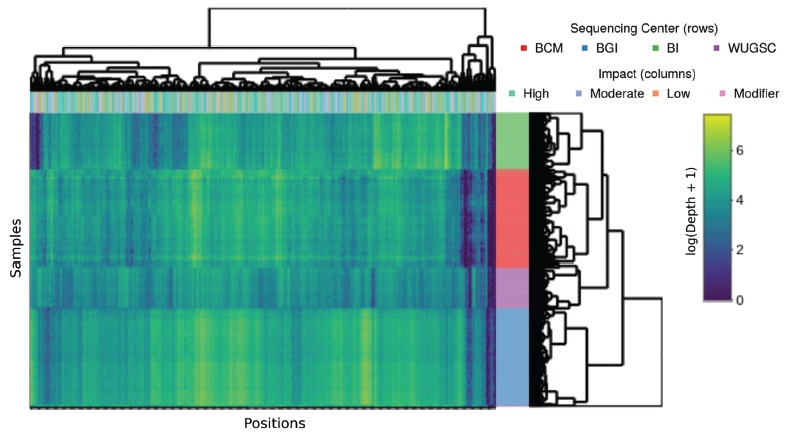
Variation in sequencing depth across sequencing centers and coding impact. Heatmap showing variation in depth across sequencing centers for the 450 variants with higher variance across samples. Each row represents a sample from one of the sequencing centers (BCM Baylor College of Medicine, BI Broad Institute, BGI, and WUGC Washington University Genome Center). 96.9% of samples are correctly assigned to their sequencing centers when considering five clusters to the dendrogram branches. The columns represent each one of the variants, with their impact classified as high, moderate, low, or modifier, which is an indicator that the coding impact does not influence the depth of coverage (p > 0.05 for each pair comparisons). An interactive version of this figure is available as File S4.

Furthermore, by analyzing the distribution of the sequencing depth of the 450 variants with higher variance, we could correctly assign 96.9% of the samples to their sequencing center when considering five clusters to the dendrogram branches depicted in Figure 2 and File S3. This finding also supports the existence of different coverage patterns for each sequencing center, evidenced in the individual coverage of each of the samples considered in these analyses.

## Discussion

Understanding how the depth of sequencing varies in sequencing experiments is essential to find a balance between the number of reads necessary to answer a genetic question and the costs and efforts required to do so (Sims *et al.*, 2014; Meienberg *et al.*, 2015; Lek *et al.*, 2016). The use of WES over WGS reduces the broad genomic region to be analyzed, dropping costs and allowing it to be more widely used in medical practice (Hu *et al.*, 2017; Manrai *et al.*, 2018; Suwinski *et al.*, 2019). The public availability of data from large genomic projects performed by worldwide consortia, such as ExAC, ESP, 1000 Genomes Project, UK10K, and GoNL, is of the utmost importance for both research and medical applications of these technologies (van Rooij *et al.*, 2017). However, one should consider the existence of methodological covariates that may introduce potential bias into the sequencing data. In our case, the possible false-negatives, which could, for example, mask the allelic frequency of a given variant returned from a sequencing center. Thus, we note the possibility of considering certain variants as “false-rares,” since their frequency would be diminished in the variant discovery process (Schaid *et al.*, 2018).

Kong *et al.* (2018) argue that both researchers and patients could benefit from clearer methodological specifications from vendors. We agree and believe that initiatives that propose the public availability of data should also provide as many technical informaion as possible. This could help users to evaluate better any bias related to the technique or methodology used to generate or to interpret the data, which could lead to erroneous or discordant clinical interpretations, for example. Here, we focused on variants that are likely to have clinical significance (comprising of 4,543 variants), since they were predicted to promote mRNA changes and/or protein structure and function alterations related to a phenotype described in ClinVar (File S2). By doing so, we aimed to assess the potential impact of variability in sequencing depth on genetic diagnosis performed by WES. This is especially relevant when a diagnostic test fails to report a variant since this could indicate either a true negative, when the genomic position of the variant is adequately captured and sequenced or a false negative when the variant is not captured or appropriately sequenced (Patwardhan *et al.*, 2015; Shigemizu *et al.*, 2015; Karlsson Linnér *et al.*, 2019).

Our results indicate that the distribution of sequencing depth varied across different sequencing centers from the 1000 Genomes Project, phase 3 (pairwise comparisons, p < 0.001). Most importantly, we found that there is a pattern of distribution in sequencing depth, which is specific to each facility (Figure 1). These findings are evidenced by the clustering of samples by PCA (69% of variance explained) and clustering of more than 95% of the samples to their sequencing centers when considering sites with highly variant coverage. These findings indicate that these patterns may be related to the methodologies used by each center. It is certainly likely that there are specific regions that differentially failed to generate adequate coverage, either due to design or capture efficiency (Altmüller *et al.*, 2016; García-García *et al.*, 2016). That means that a variant could be missed in any specific patient who was sequenced using a certain methodology specific to the sequencing center where the experiment was conducted, generating a serious problem imposed on clinical sequencing. One other piece of evidence that corroborates this is the wide standard deviation found for each of the sequencing facilities, indicating an unspecific capture reaction. The inconsistency in the breadth and depth across the targets comprising of the medically relevant variants demands the attention of professionals and patients seeking diagnosis by WES. Such an example happens with the establishment of the expanded or clinical exome capturing kits, which tend to maximize variant discovery resolution, but potentializes capture bias as well (Shamseldin *et al.*, 2017; Suwinski *et al.*, 2019). This finding also raises questions about the low frequency of a given variant that may be due to the methodological bias described in this work.

When performing WES, a critical experimental step is the capture reaction. It is well known that the efficiency of capture depends on several experimental procedures as well as on probe design, which may directly affect sequence depth and uniformity (Do *et al.*, 2012; Chandler *et al.*, 2016). Therefore, problems in the capturing reaction directly affect the final experiment results, yielding not only regions with different average depths but also leading to regions with no coverage at all (Lionel *et al.*, 2018; Wang *et al.*, 2018). We demonstrated here that differences, most likely attributed to the different methods used by the sequencing centers, proved to play a significant role in determining the distribution of sequencing depth in WES data from the 1000 Genomes Project. We understand that the methodological variability in the 1000 Genome Consortium could be the best way to achieve a more in-depth and broader variant catalog capable of establishing the bases to understand population allele frequency; however, it is also important to recognize the limitations imposed by the methods used. This finding represents a challenge for large or long-term exome sequencing projects that expect to aggregate advancements in capture techniques over time (McCarthy and MacArthur, 2017; Sanghvi *et al.*, 2018). In addition, it poses questions about the reproducibility of results among different diagnostic laboratories performing WES, indicating the need for further discussion about the use of clear open methods (both from the wet and dry lab), which could minimize such bias (Eberle *et al.*, 2017; Haga, 2017; Roy *et al.*, 2018). The proposal of returning information not only on the variants identified but also about the methods used, including the regions analyzed and all the characteristics of the sequencing reaction, could minimize misinterpretation, which directly influences the accuracy of genetic testing.

## Conclusions

Our results indicate that the sequencing depth in WES varies significantly across different facilities, leading to a systematic bias, which is most likely introduced by technical differences. Our findings indicate that the low coverage or lack of consistency between WES methodologies has direct clinical applications. It may introduce false-negatives into experiments performed for diagnostic purposes and results in variants with a lower frequency than expected. Our results are not surprising, given that the initial step for a WES experiment is the capture of the target regions to be subsequently enriched and sequenced and that this step is susceptible to the effects of many technical factors. Although difficult to address, the issue of standardized and open methodologies should be further discussed.

## Supplementary material

The following online material is available for this article:

Table S1. Detailed information on the public data we used from the 1000 Genomes Project Consortium (doc).

File S2. Variant calling file containing 4,543 variants from ClinVar (vcf). We extracted 282,453 variants from ClinVar (built 20170801, GRCh37.p13) and performed variant annotation using the Ensembl Variant Effect Predictor (VEP version 84) using the default parameters. Four thousand five hundred forty-three variants were classified as exonic and had a predicted impact on function (121 were classified as high, 2,166 moderate, 1,641 low, and 615 as a modifier).

File S3. Distribution of depth and PCA analysis for different sequencing centers, as depicted in Figure 1 (HTML). Figure 1A shows a complete distribution of depth of sequencing and an interactive 3D version of Figure 1B. Better visualized in Google Chrome.

File S4. Variation in depth across sequencing centers and coding impact data from Figure 2 (HTML). Heatmap showing the variation of depth across sequencing centers of the 450 variants with higher variance. Each row represents a sample from one of the sequencing centers (BCM Baylor College of Medicine, BI Broad Institute, BGI, and WUGC Washington University Genome Center). The columns represent each one of the variants, with their impact classified as high, moderate, low, or modifier. Better visualized in Google Chrome.
