# Supplementary material for: Methodological differences can affect sequencing depth with a possible impact on the accuracy of genetic diagnosis
Source: Genet Mol Biol. 2020 Apr 27;43(2):e20190270. doi: 10.1590/1678-4685-GMB-2019-0270 (PMC7198014; doi:10.1590/1678-4685-GMB-2019-0270)
Supplement: Table S1. [file 1415-4757-GMB-43-2-e20190270-s1.pdf]

Supplementary Material to "Methodological differences can affect sequencing depth with a possible impact on the accuracy of genetic diagnosis"

Table S1. Detailed information on the public data we used from the 1000 Genomes Project Consortium (doc).[1]

BAM FILE	SAMPLE	POPULATION	SEQUENCING CENTER
HG00097.mapped.ILLUMINA.bwa.GBR.exome.20130415.bam	HG00097	GBR	BCM
HG00099.mapped.ILLUMINA.bwa.GBR.exome.20130415.bam	HG00099	GBR	BCM
HG00105.mapped.ILLUMINA.bwa.GBR.exome.20130415.bam	HG00105	GBR	BCM
HG00106.mapped.ILLUMINA.bwa.GBR.exome.20130415.bam	HG00106	GBR	BCM
HG00112.mapped.ILLUMINA.bwa.GBR.exome.20130415.bam	HG00112	GBR	BCM
HG00115.mapped.ILLUMINA.bwa.GBR.exome.20130415.bam	HG00115	GBR	BCM
HG00118.mapped.ILLUMINA.bwa.GBR.exome.20130415.bam	HG00118	GBR	BCM
HG00122.mapped.ILLUMINA.bwa.GBR.exome.20130415.bam	HG00122	GBR	BCM
HG00126.mapped.ILLUMINA.bwa.GBR.exome.20130415.bam	HG00126	GBR	BCM
HG00128.mapped.ILLUMINA.bwa.GBR.exome.20130415.bam	HG00128	GBR	BCM
HG00129.mapped.ILLUMINA.bwa.GBR.exome.20130415.bam	HG00129	GBR	BCM
HG00130.mapped.ILLUMINA.bwa.GBR.exome.20130415.bam	HG00130	GBR	BCM
HG00132.mapped.ILLUMINA.bwa.GBR.exome.20130415.bam	HG00132	GBR	BCM
HG00141.mapped.ILLUMINA.bwa.GBR.exome.20130415.bam	HG00141	GBR	BCM
HG00142.mapped.ILLUMINA.bwa.GBR.exome.20130415.bam	HG00142	GBR	BCM
HG00143.mapped.ILLUMINA.bwa.GBR.exome.20130415.bam	HG00143	GBR	BCM
HG00148.mapped.ILLUMINA.bwa.GBR.exome.20130415.bam	HG00148	GBR	BCM

[1] This includes information on 1,112 WES samples, all sequenced in an Illumina HiSeq 2000 and belonging to any of the four sequencing centers included in this study (BCM - Baylor College of Medicine, BI - Broad Institute, BGI, and WUGC - Washington University Genome Center).

HG00149.mapped.ILLUMINA.bwa.GBR.exome.20130415.bam	HG00149	GBR	BCM
HG00150.mapped.ILLUMINA.bwa.GBR.exome.20130415.bam	HG00150	GBR	BCM
HG00151.mapped.ILLUMINA.bwa.GBR.exome.20130415.bam	HG00151	GBR	BCM
HG00173.mapped.ILLUMINA.bwa.FIN.exome.20130415.bam	HG00173	FIN	BCM
HG00177.mapped.ILLUMINA.bwa.FIN.exome.20130415.bam	HG00177	FIN	BCM
HG00179.mapped.ILLUMINA.bwa.FIN.exome.20130415.bam	HG00179	FIN	BCM
HG00180.mapped.ILLUMINA.bwa.FIN.exome.20130415.bam	HG00180	FIN	BCM
HG00231.mapped.ILLUMINA.bwa.GBR.exome.20130415.bam	HG00231	GBR	BCM
HG00234.mapped.ILLUMINA.bwa.GBR.exome.20130415.bam	HG00234	GBR	BCM
HG00235.mapped.ILLUMINA.bwa.GBR.exome.20130415.bam	HG00235	GBR	BCM
HG00266.mapped.ILLUMINA.bwa.FIN.exome.20130415.bam	HG00266	FIN	BCM
HG00267.mapped.ILLUMINA.bwa.FIN.exome.20130415.bam	HG00267	FIN	BCM
HG00269.mapped.ILLUMINA.bwa.FIN.exome.20130415.bam	HG00269	FIN	BCM
HG00288.mapped.ILLUMINA.bwa.FIN.exome.20130415.bam	HG00288	FIN	BGI
HG00290.mapped.ILLUMINA.bwa.FIN.exome.20130415.bam	HG00290	FIN	BGI
HG00304.mapped.ILLUMINA.bwa.FIN.exome.20130415.bam	HG00304	FIN	WUGSC
HG00349.mapped.ILLUMINA.bwa.FIN.exome.20130415.bam	HG00349	FIN	BCM
HG00350.mapped.ILLUMINA.bwa.FIN.exome.20130415.bam	HG00350	FIN	BCM
HG00351.mapped.ILLUMINA.bwa.FIN.exome.20130415.bam	HG00351	FIN	BCM
HG00355.mapped.ILLUMINA.bwa.FIN.exome.20130415.bam	HG00355	FIN	BCM
HG00356.mapped.ILLUMINA.bwa.FIN.exome.20130415.bam	HG00356	FIN	BCM
HG00358.mapped.ILLUMINA.bwa.FIN.exome.20130415.bam	HG00358	FIN	BCM
HG00360.mapped.ILLUMINA.bwa.FIN.exome.20130415.bam	HG00360	FIN	BCM
HG00362.mapped.ILLUMINA.bwa.FIN.exome.20130415.bam	HG00362	FIN	BCM
HG00364.mapped.ILLUMINA.bwa.FIN.exome.20130415.bam	HG00364	FIN	BCM
HG00365.mapped.ILLUMINA.bwa.FIN.exome.20130415.bam	HG00365	FIN	BCM
HG00409.mapped.ILLUMINA.bwa.CHS.exome.20130415.bam	HG00409	CHS	BI
HG00559.mapped.ILLUMINA.bwa.CHS.exome.20130415.bam	HG00559	CHS	BCM
HG00560.mapped.ILLUMINA.bwa.CHS.exome.20130415.bam	HG00560	CHS	BCM
HG00565.mapped.ILLUMINA.bwa.CHS.exome.20130415.bam	HG00565	CHS	BCM

HG00566.mapped.ILLUMINA.bwa.CHS.exome.20130415.bam	HG00566	CHS	BCM
HG00592.mapped.ILLUMINA.bwa.CHS.exome.20130415.bam	HG00592	CHS	BCM
HG00593.mapped.ILLUMINA.bwa.CHS.exome.20130415.bam	HG00593	CHS	BCM
HG00595.mapped.ILLUMINA.bwa.CHS.exome.20130415.bam	HG00595	CHS	BCM
HG00596.mapped.ILLUMINA.bwa.CHS.exome.20130415.bam	HG00596	CHS	BCM
HG00622.mapped.ILLUMINA.bwa.CHS.exome.20130415.bam	HG00622	CHS	BI
HG00717.mapped.ILLUMINA.bwa.CHS.exome.20130415.bam	HG00717	CHS	WUGSC
HG00742.mapped.ILLUMINA.bwa.PUR.exome.20130415.bam	HG00742	PUR	WUGSC
HG00743.mapped.ILLUMINA.bwa.PUR.exome.20130415.bam	HG00743	PUR	WUGSC
HG00759.mapped.ILLUMINA.bwa.CDX.exome.20130415.bam	HG00759	CDX	BGI
HG00766.mapped.ILLUMINA.bwa.CDX.exome.20130415.bam	HG00766	CDX	BGI
HG00867.mapped.ILLUMINA.bwa.CDX.exome.20130415.bam	HG00867	CDX	BGI
HG01058.mapped.ILLUMINA.bwa.PUR.exome.20130415.bam	HG01058	PUR	WUGSC
HG01063.mapped.ILLUMINA.bwa.PUR.exome.20130415.bam	HG01063	PUR	WUGSC
HG01064.mapped.ILLUMINA.bwa.PUR.exome.20130415.bam	HG01064	PUR	WUGSC
HG01077.mapped.ILLUMINA.bwa.PUR.exome.20130415.bam	HG01077	PUR	WUGSC
HG01088.mapped.ILLUMINA.bwa.PUR.exome.20130415.bam	HG01088	PUR	WUGSC
HG01089.mapped.ILLUMINA.bwa.PUR.exome.20130415.bam	HG01089	PUR	WUGSC
HG01092.mapped.ILLUMINA.bwa.PUR.exome.20130415.bam	HG01092	PUR	WUGSC
HG01119.mapped.ILLUMINA.bwa.CLM.exome.20130415.bam	HG01119	CLM	BGI
HG01121.mapped.ILLUMINA.bwa.CLM.exome.20130415.bam	HG01121	CLM	BGI
HG01122.mapped.ILLUMINA.bwa.CLM.exome.20130415.bam	HG01122	CLM	BGI
HG01124.mapped.ILLUMINA.bwa.CLM.exome.20130415.bam	HG01124	CLM	BCM
HG01125.mapped.ILLUMINA.bwa.CLM.exome.20130415.bam	HG01125	CLM	BCM
HG01130.mapped.ILLUMINA.bwa.CLM.exome.20130415.bam	HG01130	CLM	BGI
HG01131.mapped.ILLUMINA.bwa.CLM.exome.20130415.bam	HG01131	CLM	BGI
HG01133.mapped.ILLUMINA.bwa.CLM.exome.20130415.bam	HG01133	CLM	BCM
HG01134.mapped.ILLUMINA.bwa.CLM.exome.20130415.bam	HG01134	CLM	BCM
HG01136.mapped.ILLUMINA.bwa.CLM.exome.20130415.bam	HG01136	CLM	BCM
HG01137.mapped.ILLUMINA.bwa.CLM.exome.20130415.bam	HG01137	CLM	BCM

HG01139.mapped.ILLUMINA.bwa.CLM.exome.20130415.bam	HG01139	CLM	BCM
HG01140.mapped.ILLUMINA.bwa.CLM.exome.20130415.bam	HG01140	CLM	BCM
HG01142.mapped.ILLUMINA.bwa.CLM.exome.20130415.bam	HG01142	CLM	BGI
HG01148.mapped.ILLUMINA.bwa.CLM.exome.20130415.bam	HG01148	CLM	BCM
HG01149.mapped.ILLUMINA.bwa.CLM.exome.20130415.bam	HG01149	CLM	BCM
HG01161.mapped.ILLUMINA.bwa.PUR.exome.20130415.bam	HG01161	PUR	WUGSC
HG01162.mapped.ILLUMINA.bwa.PUR.exome.20130415.bam	HG01162	PUR	WUGSC
HG01164.mapped.ILLUMINA.bwa.PUR.exome.20130415.bam	HG01164	PUR	WUGSC
HG01200.mapped.ILLUMINA.bwa.PUR.exome.20130415.bam	HG01200	PUR	WUGSC
HG01269.mapped.ILLUMINA.bwa.CLM.exome.20130415.bam	HG01269	CLM	BGI
HG01271.mapped.ILLUMINA.bwa.CLM.exome.20130415.bam	HG01271	CLM	BCM
HG01272.mapped.ILLUMINA.bwa.CLM.exome.20130415.bam	HG01272	CLM	BCM
HG01275.mapped.ILLUMINA.bwa.CLM.exome.20130415.bam	HG01275	CLM	BCM
HG01277.mapped.ILLUMINA.bwa.CLM.exome.20130415.bam	HG01277	CLM	BCM
HG01280.mapped.ILLUMINA.bwa.CLM.exome.20130415.bam	HG01280	CLM	BGI
HG01281.mapped.ILLUMINA.bwa.CLM.exome.20130415.bam	HG01281	CLM	BGI
HG01284.mapped.ILLUMINA.bwa.CLM.exome.20130415.bam	HG01284	CLM	BGI
HG01286.mapped.ILLUMINA.bwa.PUR.exome.20130415.bam	HG01286	PUR	WUGSC
HG01302.mapped.ILLUMINA.bwa.PUR.exome.20130415.bam	HG01302	PUR	WUGSC
HG01303.mapped.ILLUMINA.bwa.PUR.exome.20130415.bam	HG01303	PUR	WUGSC
HG01305.mapped.ILLUMINA.bwa.PUR.exome.20130415.bam	HG01305	PUR	WUGSC
HG01308.mapped.ILLUMINA.bwa.PUR.exome.20130415.bam	HG01308	PUR	WUGSC
HG01311.mapped.ILLUMINA.bwa.PUR.exome.20130415.bam	HG01311	PUR	WUGSC
HG01312.mapped.ILLUMINA.bwa.PUR.exome.20130415.bam	HG01312	PUR	WUGSC
HG01323.mapped.ILLUMINA.bwa.PUR.exome.20130415.bam	HG01323	PUR	WUGSC
HG01325.mapped.ILLUMINA.bwa.PUR.exome.20130415.bam	HG01325	PUR	WUGSC
HG01326.mapped.ILLUMINA.bwa.PUR.exome.20130415.bam	HG01326	PUR	WUGSC
HG01344.mapped.ILLUMINA.bwa.CLM.exome.20130415.bam	HG01344	CLM	BCM
HG01345.mapped.ILLUMINA.bwa.CLM.exome.20130415.bam	HG01345	CLM	BCM
HG01348.mapped.ILLUMINA.bwa.CLM.exome.20130415.bam	HG01348	CLM	BCM

HG01356.mapped.ILLUMINA.bwa.CLM.exome.20130415.bam	HG01356	CLM	BCM
HG01357.mapped.ILLUMINA.bwa.CLM.exome.20130415.bam	HG01357	CLM	BCM
HG01362.mapped.ILLUMINA.bwa.CLM.exome.20130415.bam	HG01362	CLM	BGI
HG01363.mapped.ILLUMINA.bwa.CLM.exome.20130415.bam	HG01363	CLM	BGI
HG01369.mapped.ILLUMINA.bwa.CLM.exome.20130415.bam	HG01369	CLM	BGI
HG01372.mapped.ILLUMINA.bwa.CLM.exome.20130415.bam	HG01372	CLM	BGI
HG01392.mapped.ILLUMINA.bwa.PUR.exome.20130415.bam	HG01392	PUR	BGI
HG01393.mapped.ILLUMINA.bwa.PUR.exome.20130415.bam	HG01393	PUR	BGI
HG01395.mapped.ILLUMINA.bwa.PUR.exome.20130415.bam	HG01395	PUR	BGI
HG01396.mapped.ILLUMINA.bwa.PUR.exome.20130415.bam	HG01396	PUR	BGI
HG01398.mapped.ILLUMINA.bwa.PUR.exome.20130415.bam	HG01398	PUR	WUGSC
HG01402.mapped.ILLUMINA.bwa.PUR.exome.20130415.bam	HG01402	PUR	BGI
HG01403.mapped.ILLUMINA.bwa.PUR.exome.20130415.bam	HG01403	PUR	BGI
HG01405.mapped.ILLUMINA.bwa.PUR.exome.20130415.bam	HG01405	PUR	BGI
HG01412.mapped.ILLUMINA.bwa.PUR.exome.20130415.bam	HG01412	PUR	BGI
HG01413.mapped.ILLUMINA.bwa.PUR.exome.20130415.bam	HG01413	PUR	BGI
HG01414.mapped.ILLUMINA.bwa.PUR.exome.20130415.bam	HG01414	PUR	BGI
HG01431.mapped.ILLUMINA.bwa.CLM.exome.20130415.bam	HG01431	CLM	BGI
HG01432.mapped.ILLUMINA.bwa.CLM.exome.20130415.bam	HG01432	CLM	BGI
HG01435.mapped.ILLUMINA.bwa.CLM.exome.20130415.bam	HG01435	CLM	BGI
HG01443.mapped.ILLUMINA.bwa.CLM.exome.20130415.bam	HG01443	CLM	BGI
HG01444.mapped.ILLUMINA.bwa.CLM.exome.20130415.bam	HG01444	CLM	BGI
HG01447.mapped.ILLUMINA.bwa.CLM.exome.20130415.bam	HG01447	CLM	BGI
HG01459.mapped.ILLUMINA.bwa.CLM.exome.20130415.bam	HG01459	CLM	BGI
HG01468.mapped.ILLUMINA.bwa.CLM.exome.20130415.bam	HG01468	CLM	WUGSC
HG01474.mapped.ILLUMINA.bwa.CLM.exome.20130415.bam	HG01474	CLM	WUGSC
HG01479.mapped.ILLUMINA.bwa.CLM.exome.20130415.bam	HG01479	CLM	WUGSC
HG01485.mapped.ILLUMINA.bwa.CLM.exome.20130415.bam	HG01485	CLM	BGI
HG01486.mapped.ILLUMINA.bwa.CLM.exome.20130415.bam	HG01486	CLM	BGI
HG01500.mapped.ILLUMINA.bwa.IBS.exome.20130415.bam	HG01500	IBS	BI

HG01501.mapped.ILLUMINA.bwa.IBS.exome.20130415.bam	HG01501	IBS	BCM
HG01503.mapped.ILLUMINA.bwa.IBS.exome.20130415.bam	HG01503	IBS	BCM
HG01504.mapped.ILLUMINA.bwa.IBS.exome.20130415.bam	HG01504	IBS	BCM
HG01556.mapped.ILLUMINA.bwa.CLM.exome.20130415.bam	HG01556	CLM	BGI
HG01565.mapped.ILLUMINA.bwa.PEL.exome.20130415.bam	HG01565	PEL	BI
HG01566.mapped.ILLUMINA.bwa.PEL.exome.20130415.bam	HG01566	PEL	BCM
HG01571.mapped.ILLUMINA.bwa.PEL.exome.20130415.bam	HG01571	PEL	BCM
HG01572.mapped.ILLUMINA.bwa.PEL.exome.20130415.bam	HG01572	PEL	BCM
HG01583.mapped.ILLUMINA.bwa.PJL.exome.20130415.bam	HG01583	PJL	BI
HG01586.mapped.ILLUMINA.bwa.PJL.exome.20130415.bam	HG01586	PJL	WUGSC
HG01589.mapped.ILLUMINA.bwa.PJL.exome.20130415.bam	HG01589	PJL	WUGSC
HG01593.mapped.ILLUMINA.bwa.PJL.exome.20130415.bam	HG01593	PJL	WUGSC
HG01602.mapped.ILLUMINA.bwa.IBS.exome.20130415.bam	HG01602	IBS	BCM
HG01603.mapped.ILLUMINA.bwa.IBS.exome.20130415.bam	HG01603	IBS	BCM
HG01605.mapped.ILLUMINA.bwa.IBS.exome.20130415.bam	HG01605	IBS	BCM
HG01606.mapped.ILLUMINA.bwa.IBS.exome.20130415.bam	HG01606	IBS	BCM
HG01607.mapped.ILLUMINA.bwa.IBS.exome.20130415.bam	HG01607	IBS	BCM
HG01608.mapped.ILLUMINA.bwa.IBS.exome.20130415.bam	HG01608	IBS	BCM
HG01610.mapped.ILLUMINA.bwa.IBS.exome.20130415.bam	HG01610	IBS	BCM
HG01612.mapped.ILLUMINA.bwa.IBS.exome.20130415.bam	HG01612	IBS	BCM
HG01613.mapped.ILLUMINA.bwa.IBS.exome.20130415.bam	HG01613	IBS	BCM
HG01615.mapped.ILLUMINA.bwa.IBS.exome.20130415.bam	HG01615	IBS	BCM
HG01617.mapped.ILLUMINA.bwa.IBS.exome.20130415.bam	HG01617	IBS	BCM
HG01618.mapped.ILLUMINA.bwa.IBS.exome.20130415.bam	HG01618	IBS	BCM
HG01619.mapped.ILLUMINA.bwa.IBS.exome.20130415.bam	HG01619	IBS	BCM
HG01620.mapped.ILLUMINA.bwa.IBS.exome.20130415.bam	HG01620	IBS	BCM
HG01623.mapped.ILLUMINA.bwa.IBS.exome.20130415.bam	HG01623	IBS	BCM
HG01624.mapped.ILLUMINA.bwa.IBS.exome.20130415.bam	HG01624	IBS	BCM
HG01625.mapped.ILLUMINA.bwa.IBS.exome.20130415.bam	HG01625	IBS	BCM
HG01626.mapped.ILLUMINA.bwa.IBS.exome.20130415.bam	HG01626	IBS	BCM

HG01628.mapped.ILLUMINA.bwa.IBS.exome.20130415.bam	HG01628	IBS	BCM
HG01630.mapped.ILLUMINA.bwa.IBS.exome.20130415.bam	HG01630	IBS	BCM
HG01668.mapped.ILLUMINA.bwa.IBS.exome.20130415.bam	HG01668	IBS	BCM
HG01669.mapped.ILLUMINA.bwa.IBS.exome.20130415.bam	HG01669	IBS	BCM
HG01670.mapped.ILLUMINA.bwa.IBS.exome.20130415.bam	HG01670	IBS	BCM
HG01672.mapped.ILLUMINA.bwa.IBS.exome.20130415.bam	HG01672	IBS	BCM
HG01765.mapped.ILLUMINA.bwa.IBS.exome.20130415.bam	HG01765	IBS	BGI
HG01766.mapped.ILLUMINA.bwa.IBS.exome.20130415.bam	HG01766	IBS	BGI
HG01767.mapped.ILLUMINA.bwa.IBS.exome.20130415.bam	HG01767	IBS	BGI
HG01768.mapped.ILLUMINA.bwa.IBS.exome.20130415.bam	HG01768	IBS	BGI
HG01770.mapped.ILLUMINA.bwa.IBS.exome.20130415.bam	HG01770	IBS	BCM
HG01771.mapped.ILLUMINA.bwa.IBS.exome.20130415.bam	HG01771	IBS	BCM
HG01773.mapped.ILLUMINA.bwa.IBS.exome.20130415.bam	HG01773	IBS	BCM
HG01775.mapped.ILLUMINA.bwa.IBS.exome.20130415.bam	HG01775	IBS	BCM
HG01776.mapped.ILLUMINA.bwa.IBS.exome.20130415.bam	HG01776	IBS	BCM
HG01777.mapped.ILLUMINA.bwa.IBS.exome.20130415.bam	HG01777	IBS	BCM
HG01779.mapped.ILLUMINA.bwa.IBS.exome.20130415.bam	HG01779	IBS	BCM
HG01781.mapped.ILLUMINA.bwa.IBS.exome.20130415.bam	HG01781	IBS	BCM
HG01783.mapped.ILLUMINA.bwa.IBS.exome.20130415.bam	HG01783	IBS	BCM
HG01784.mapped.ILLUMINA.bwa.IBS.exome.20130415.bam	HG01784	IBS	BCM
HG01785.mapped.ILLUMINA.bwa.IBS.exome.20130415.bam	HG01785	IBS	WUGSC
HG01786.mapped.ILLUMINA.bwa.IBS.exome.20130415.bam	HG01786	IBS	WUGSC
HG01789.mapped.ILLUMINA.bwa.GBR.exome.20130415.bam	HG01789	GBR	BCM
HG01790.mapped.ILLUMINA.bwa.GBR.exome.20130415.bam	HG01790	GBR	BCM
HG01889.mapped.ILLUMINA.bwa.ACB.exome.20130415.bam	HG01889	ACB	BCM
HG01890.mapped.ILLUMINA.bwa.ACB.exome.20130415.bam	HG01890	ACB	BCM
HG01894.mapped.ILLUMINA.bwa.ACB.exome.20130415.bam	HG01894	ACB	BCM
HG01912.mapped.ILLUMINA.bwa.ACB.exome.20130415.bam	HG01912	ACB	BCM
HG01961.mapped.ILLUMINA.bwa.PEL.exome.20130415.bam	HG01961	PEL	WUGSC
HG01965.mapped.ILLUMINA.bwa.PEL.exome.20130415.bam	HG01965	PEL	WUGSC

HG01979.mapped.ILLUMINA.bwa.PEL.exome.20130415.bam	HG01979	PEL	BCM
HG01980.mapped.ILLUMINA.bwa.PEL.exome.20130415.bam	HG01980	PEL	BCM
HG01991.mapped.ILLUMINA.bwa.PEL.exome.20130415.bam	HG01991	PEL	BCM
HG01992.mapped.ILLUMINA.bwa.PEL.exome.20130415.bam	HG01992	PEL	BCM
HG01997.mapped.ILLUMINA.bwa.PEL.exome.20130415.bam	HG01997	PEL	BCM
HG02002.mapped.ILLUMINA.bwa.PEL.exome.20130415.bam	HG02002	PEL	BCM
HG02003.mapped.ILLUMINA.bwa.PEL.exome.20130415.bam	HG02003	PEL	BCM
HG02006.mapped.ILLUMINA.bwa.PEL.exome.20130415.bam	HG02006	PEL	BGI
HG02008.mapped.ILLUMINA.bwa.PEL.exome.20130415.bam	HG02008	PEL	BCM
HG02009.mapped.ILLUMINA.bwa.ACB.exome.20130415.bam	HG02009	ACB	BCM
HG02010.mapped.ILLUMINA.bwa.ACB.exome.20130415.bam	HG02010	ACB	BCM
HG02016.mapped.ILLUMINA.bwa.KHV.exome.20130415.bam	HG02016	KHV	BCM
HG02017.mapped.ILLUMINA.bwa.KHV.exome.20130415.bam	HG02017	KHV	BCM
HG02019.mapped.ILLUMINA.bwa.KHV.exome.20130415.bam	HG02019	KHV	BCM
HG02020.mapped.ILLUMINA.bwa.KHV.exome.20130415.bam	HG02020	KHV	BCM
HG02035.mapped.ILLUMINA.bwa.KHV.exome.20130415.bam	HG02035	KHV	BCM
HG02054.mapped.ILLUMINA.bwa.ACB.exome.20130415.bam	HG02054	ACB	BCM
HG02075.mapped.ILLUMINA.bwa.KHV.exome.20130415.bam	HG02075	KHV	BCM
HG02076.mapped.ILLUMINA.bwa.KHV.exome.20130415.bam	HG02076	KHV	BCM
HG02078.mapped.ILLUMINA.bwa.KHV.exome.20130415.bam	HG02078	KHV	BCM
HG02079.mapped.ILLUMINA.bwa.KHV.exome.20130415.bam	HG02079	KHV	BCM
HG02081.mapped.ILLUMINA.bwa.KHV.exome.20130415.bam	HG02081	KHV	BCM
HG02082.mapped.ILLUMINA.bwa.KHV.exome.20130415.bam	HG02082	KHV	BCM
HG02084.mapped.ILLUMINA.bwa.KHV.exome.20130415.bam	HG02084	KHV	BCM
HG02085.mapped.ILLUMINA.bwa.KHV.exome.20130415.bam	HG02085	KHV	BCM
HG02086.mapped.ILLUMINA.bwa.KHV.exome.20130415.bam	HG02086	KHV	BCM
HG02087.mapped.ILLUMINA.bwa.KHV.exome.20130415.bam	HG02087	KHV	BCM
HG02088.mapped.ILLUMINA.bwa.KHV.exome.20130415.bam	HG02088	KHV	BCM
HG02089.mapped.ILLUMINA.bwa.PEL.exome.20130415.bam	HG02089	PEL	BCM
HG02090.mapped.ILLUMINA.bwa.PEL.exome.20130415.bam	HG02090	PEL	BCM

HG02102.mapped.ILLUMINA.bwa.PEL.exome.20130415.bam	HG02102	PEL	BGI
HG02104.mapped.ILLUMINA.bwa.PEL.exome.20130415.bam	HG02104	PEL	BCM
HG02105.mapped.ILLUMINA.bwa.PEL.exome.20130415.bam	HG02105	PEL	BCM
HG02113.mapped.ILLUMINA.bwa.KHV.exome.20130415.bam	HG02113	KHV	BCM
HG02121.mapped.ILLUMINA.bwa.KHV.exome.20130415.bam	HG02121	KHV	BCM
HG02122.mapped.ILLUMINA.bwa.KHV.exome.20130415.bam	HG02122	KHV	BCM
HG02127.mapped.ILLUMINA.bwa.KHV.exome.20130415.bam	HG02127	KHV	BCM
HG02128.mapped.ILLUMINA.bwa.KHV.exome.20130415.bam	HG02128	KHV	BCM
HG02138.mapped.ILLUMINA.bwa.KHV.exome.20130415.bam	HG02138	KHV	BCM
HG02139.mapped.ILLUMINA.bwa.KHV.exome.20130415.bam	HG02139	KHV	BCM
HG02140.mapped.ILLUMINA.bwa.KHV.exome.20130415.bam	HG02140	KHV	BCM
HG02141.mapped.ILLUMINA.bwa.KHV.exome.20130415.bam	HG02141	KHV	BCM
HG02150.mapped.ILLUMINA.bwa.PEL.exome.20130415.bam	HG02150	PEL	BGI
HG02152.mapped.ILLUMINA.bwa.CDX.exome.20130415.bam	HG02152	CDX	BCM
HG02153.mapped.ILLUMINA.bwa.CDX.exome.20130415.bam	HG02153	CDX	BCM
HG02154.mapped.ILLUMINA.bwa.CDX.exome.20130415.bam	HG02154	CDX	BCM
HG02155.mapped.ILLUMINA.bwa.CDX.exome.20130415.bam	HG02155	CDX	BCM
HG02156.mapped.ILLUMINA.bwa.CDX.exome.20130415.bam	HG02156	CDX	BCM
HG02164.mapped.ILLUMINA.bwa.CDX.exome.20130415.bam	HG02164	CDX	BCM
HG02165.mapped.ILLUMINA.bwa.CDX.exome.20130415.bam	HG02165	CDX	BCM
HG02166.mapped.ILLUMINA.bwa.CDX.exome.20130415.bam	HG02166	CDX	BCM
HG02178.mapped.ILLUMINA.bwa.CDX.exome.20130415.bam	HG02178	CDX	BCM
HG02179.mapped.ILLUMINA.bwa.CDX.exome.20130415.bam	HG02179	CDX	BCM
HG02180.mapped.ILLUMINA.bwa.CDX.exome.20130415.bam	HG02180	CDX	BCM
HG02181.mapped.ILLUMINA.bwa.CDX.exome.20130415.bam	HG02181	CDX	BCM
HG02182.mapped.ILLUMINA.bwa.CDX.exome.20130415.bam	HG02182	CDX	BCM
HG02184.mapped.ILLUMINA.bwa.CDX.exome.20130415.bam	HG02184	CDX	BCM
HG02185.mapped.ILLUMINA.bwa.CDX.exome.20130415.bam	HG02185	CDX	BCM
HG02186.mapped.ILLUMINA.bwa.CDX.exome.20130415.bam	HG02186	CDX	BCM
HG02187.mapped.ILLUMINA.bwa.CDX.exome.20130415.bam	HG02187	CDX	BCM

HG02188.mapped.ILLUMINA.bwa.CDX.exome.20130415.bam	HG02188	CDX	BCM
HG02190.mapped.ILLUMINA.bwa.CDX.exome.20130415.bam	HG02190	CDX	BCM
HG02219.mapped.ILLUMINA.bwa.IBS.exome.20130415.bam	HG02219	IBS	WUGSC
HG02220.mapped.ILLUMINA.bwa.IBS.exome.20130415.bam	HG02220	IBS	BCM
HG02221.mapped.ILLUMINA.bwa.IBS.exome.20130415.bam	HG02221	IBS	BCM
HG02235.mapped.ILLUMINA.bwa.IBS.exome.20130415.bam	HG02235	IBS	BCM
HG02236.mapped.ILLUMINA.bwa.IBS.exome.20130415.bam	HG02236	IBS	BCM
HG02252.mapped.ILLUMINA.bwa.PEL.exome.20130415.bam	HG02252	PEL	BGI
HG02253.mapped.ILLUMINA.bwa.PEL.exome.20130415.bam	HG02253	PEL	BGI
HG02255.mapped.ILLUMINA.bwa.ACB.exome.20130415.bam	HG02255	ACB	BCM
HG02256.mapped.ILLUMINA.bwa.ACB.exome.20130415.bam	HG02256	ACB	BCM
HG02262.mapped.ILLUMINA.bwa.PEL.exome.20130415.bam	HG02262	PEL	BGI
HG02272.mapped.ILLUMINA.bwa.PEL.exome.20130415.bam	HG02272	PEL	BCM
HG02277.mapped.ILLUMINA.bwa.PEL.exome.20130415.bam	HG02277	PEL	BCM
HG02278.mapped.ILLUMINA.bwa.PEL.exome.20130415.bam	HG02278	PEL	BCM
HG02281.mapped.ILLUMINA.bwa.ACB.exome.20130415.bam	HG02281	ACB	BCM
HG02282.mapped.ILLUMINA.bwa.ACB.exome.20130415.bam	HG02282	ACB	BCM
HG02285.mapped.ILLUMINA.bwa.PEL.exome.20130415.bam	HG02285	PEL	BCM
HG02286.mapped.ILLUMINA.bwa.PEL.exome.20130415.bam	HG02286	PEL	BCM
HG02301.mapped.ILLUMINA.bwa.PEL.exome.20130415.bam	HG02301	PEL	BCM
HG02304.mapped.ILLUMINA.bwa.PEL.exome.20130415.bam	HG02304	PEL	BGI
HG02312.mapped.ILLUMINA.bwa.PEL.exome.20130415.bam	HG02312	PEL	BGI
HG02317.mapped.ILLUMINA.bwa.ACB.exome.20130415.bam	HG02317	ACB	BCM
HG02318.mapped.ILLUMINA.bwa.ACB.exome.20130415.bam	HG02318	ACB	BCM
HG02345.mapped.ILLUMINA.bwa.PEL.exome.20130415.bam	HG02345	PEL	WUGSC
HG02348.mapped.ILLUMINA.bwa.PEL.exome.20130415.bam	HG02348	PEL	BGI
HG02439.mapped.ILLUMINA.bwa.ACB.exome.20130415.bam	HG02439	ACB	BGI
HG02455.mapped.ILLUMINA.bwa.ACB.exome.20130415.bam	HG02455	ACB	BGI
HG02461.mapped.ILLUMINA.bwa.GWD.exome.20130415.bam	HG02461	GWD	BI
HG02462.mapped.ILLUMINA.bwa.GWD.exome.20130415.bam	HG02462	GWD	BI

HG02464.mapped.ILLUMINA.bwa.GWD.exome.20130415.bam	HG02464	GWD	BI
HG02465.mapped.ILLUMINA.bwa.GWD.exome.20130415.bam	HG02465	GWD	BI
HG02476.mapped.ILLUMINA.bwa.ACB.exome.20130415.bam	HG02476	ACB	WUGSC
HG02477.mapped.ILLUMINA.bwa.ACB.exome.20130415.bam	HG02477	ACB	WUGSC
HG02479.mapped.ILLUMINA.bwa.ACB.exome.20130415.bam	HG02479	ACB	BCM
HG02481.mapped.ILLUMINA.bwa.ACB.exome.20130415.bam	HG02481	ACB	WUGSC
HG02484.mapped.ILLUMINA.bwa.ACB.exome.20130415.bam	HG02484	ACB	BCM
HG02485.mapped.ILLUMINA.bwa.ACB.exome.20130415.bam	HG02485	ACB	BCM
HG02491.mapped.ILLUMINA.bwa.PJL.exome.20130415.bam	HG02491	PJL	BI
HG02493.mapped.ILLUMINA.bwa.PJL.exome.20130415.bam	HG02493	PJL	BGI
HG02494.mapped.ILLUMINA.bwa.PJL.exome.20130415.bam	HG02494	PJL	BGI
HG02496.mapped.ILLUMINA.bwa.ACB.exome.20130415.bam	HG02496	ACB	BCM
HG02497.mapped.ILLUMINA.bwa.ACB.exome.20130415.bam	HG02497	ACB	BCM
HG02501.mapped.ILLUMINA.bwa.ACB.exome.20130415.bam	HG02501	ACB	WUGSC
HG02502.mapped.ILLUMINA.bwa.ACB.exome.20130415.bam	HG02502	ACB	WUGSC
HG02505.mapped.ILLUMINA.bwa.ACB.exome.20130415.bam	HG02505	ACB	WUGSC
HG02508.mapped.ILLUMINA.bwa.ACB.exome.20130415.bam	HG02508	ACB	BCM
HG02511.mapped.ILLUMINA.bwa.ACB.exome.20130415.bam	HG02511	ACB	BCM
HG02536.mapped.ILLUMINA.bwa.ACB.exome.20130415.bam	HG02536	ACB	WUGSC
HG02537.mapped.ILLUMINA.bwa.ACB.exome.20130415.bam	HG02537	ACB	BGI
HG02541.mapped.ILLUMINA.bwa.ACB.exome.20130415.bam	HG02541	ACB	BGI
HG02545.mapped.ILLUMINA.bwa.ACB.exome.20130415.bam	HG02545	ACB	WUGSC
HG02546.mapped.ILLUMINA.bwa.ACB.exome.20130415.bam	HG02546	ACB	WUGSC
HG02549.mapped.ILLUMINA.bwa.ACB.exome.20130415.bam	HG02549	ACB	BGI
HG02554.mapped.ILLUMINA.bwa.ACB.exome.20130415.bam	HG02554	ACB	BGI
HG02555.mapped.ILLUMINA.bwa.ACB.exome.20130415.bam	HG02555	ACB	BGI
HG02557.mapped.ILLUMINA.bwa.ACB.exome.20130415.bam	HG02557	ACB	WUGSC
HG02558.mapped.ILLUMINA.bwa.ACB.exome.20130415.bam	HG02558	ACB	WUGSC
HG02562.mapped.ILLUMINA.bwa.GWD.exome.20130415.bam	HG02562	GWD	BI
HG02568.mapped.ILLUMINA.bwa.GWD.exome.20130415.bam	HG02568	GWD	BGI

HG02570.mapped.ILLUMINA.bwa.GWD.exome.20130415.bam	HG02570	GWD	BI
HG02573.mapped.ILLUMINA.bwa.GWD.exome.20130415.bam	HG02573	GWD	BI
HG02577.mapped.ILLUMINA.bwa.ACB.exome.20130415.bam	HG02577	ACB	WUGSC
HG02580.mapped.ILLUMINA.bwa.ACB.exome.20130415.bam	HG02580	ACB	BGI
HG02582.mapped.ILLUMINA.bwa.GWD.exome.20130415.bam	HG02582	GWD	BI
HG02583.mapped.ILLUMINA.bwa.GWD.exome.20130415.bam	HG02583	GWD	BI
HG02588.mapped.ILLUMINA.bwa.GWD.exome.20130415.bam	HG02588	GWD	BI
HG02589.mapped.ILLUMINA.bwa.GWD.exome.20130415.bam	HG02589	GWD	BI
HG02595.mapped.ILLUMINA.bwa.GWD.exome.20130415.bam	HG02595	GWD	BI
HG02597.mapped.ILLUMINA.bwa.PJL.exome.20130415.bam	HG02597	PJL	WUGSC
HG02600.mapped.ILLUMINA.bwa.PJL.exome.20130415.bam	HG02600	PJL	BI
HG02603.mapped.ILLUMINA.bwa.PJL.exome.20130415.bam	HG02603	PJL	BI
HG02611.mapped.ILLUMINA.bwa.GWD.exome.20130415.bam	HG02611	GWD	BI
HG02613.mapped.ILLUMINA.bwa.GWD.exome.20130415.bam	HG02613	GWD	BI
HG02620.mapped.ILLUMINA.bwa.GWD.exome.20130415.bam	HG02620	GWD	BI
HG02621.mapped.ILLUMINA.bwa.GWD.exome.20130415.bam	HG02621	GWD	BI
HG02624.mapped.ILLUMINA.bwa.GWD.exome.20130415.bam	HG02624	GWD	BI
HG02629.mapped.ILLUMINA.bwa.GWD.exome.20130415.bam	HG02629	GWD	BI
HG02642.mapped.ILLUMINA.bwa.GWD.exome.20130415.bam	HG02642	GWD	BI
HG02643.mapped.ILLUMINA.bwa.GWD.exome.20130415.bam	HG02643	GWD	BI
HG02646.mapped.ILLUMINA.bwa.GWD.exome.20130415.bam	HG02646	GWD	BI
HG02648.mapped.ILLUMINA.bwa.PJL.exome.20130415.bam	HG02648	PJL	BGI
HG02649.mapped.ILLUMINA.bwa.PJL.exome.20130415.bam	HG02649	PJL	BGI
HG02651.mapped.ILLUMINA.bwa.PJL.exome.20130415.bam	HG02651	PJL	BGI
HG02652.mapped.ILLUMINA.bwa.PJL.exome.20130415.bam	HG02652	PJL	BGI
HG02658.mapped.ILLUMINA.bwa.PJL.exome.20130415.bam	HG02658	PJL	BI
HG02661.mapped.ILLUMINA.bwa.PJL.exome.20130415.bam	HG02661	PJL	BI
HG02666.mapped.ILLUMINA.bwa.GWD.exome.20130415.bam	HG02666	GWD	BI
HG02675.mapped.ILLUMINA.bwa.GWD.exome.20130415.bam	HG02675	GWD	BI
HG02676.mapped.ILLUMINA.bwa.GWD.exome.20130415.bam	HG02676	GWD	BI

HG02678.mapped.ILLUMINA.bwa.GWD.exome.20130415.bam	HG02678	GWD	BI
HG02679.mapped.ILLUMINA.bwa.GWD.exome.20130415.bam	HG02679	GWD	BI
HG02681.mapped.ILLUMINA.bwa.PJL.exome.20130415.bam	HG02681	PJL	BGI
HG02682.mapped.ILLUMINA.bwa.PJL.exome.20130415.bam	HG02682	PJL	BGI
HG02688.mapped.ILLUMINA.bwa.PJL.exome.20130415.bam	HG02688	PJL	BI
HG02690.mapped.ILLUMINA.bwa.PJL.exome.20130415.bam	HG02690	PJL	BGI
HG02691.mapped.ILLUMINA.bwa.PJL.exome.20130415.bam	HG02691	PJL	BGI
HG02694.mapped.ILLUMINA.bwa.PJL.exome.20130415.bam	HG02694	PJL	WUGSC
HG02699.mapped.ILLUMINA.bwa.PJL.exome.20130415.bam	HG02699	PJL	BGI
HG02700.mapped.ILLUMINA.bwa.PJL.exome.20130415.bam	HG02700	PJL	BGI
HG02702.mapped.ILLUMINA.bwa.GWD.exome.20130415.bam	HG02702	GWD	BI
HG02703.mapped.ILLUMINA.bwa.GWD.exome.20130415.bam	HG02703	GWD	BI
HG02716.mapped.ILLUMINA.bwa.GWD.exome.20130415.bam	HG02716	GWD	BI
HG02727.mapped.ILLUMINA.bwa.PJL.exome.20130415.bam	HG02727	PJL	BI
HG02731.mapped.ILLUMINA.bwa.PJL.exome.20130415.bam	HG02731	PJL	WUGSC
HG02733.mapped.ILLUMINA.bwa.PJL.exome.20130415.bam	HG02733	PJL	BI
HG02736.mapped.ILLUMINA.bwa.PJL.exome.20130415.bam	HG02736	PJL	BGI
HG02737.mapped.ILLUMINA.bwa.PJL.exome.20130415.bam	HG02737	PJL	BGI
HG02756.mapped.ILLUMINA.bwa.GWD.exome.20130415.bam	HG02756	GWD	BI
HG02759.mapped.ILLUMINA.bwa.GWD.exome.20130415.bam	HG02759	GWD	BGI
HG02760.mapped.ILLUMINA.bwa.GWD.exome.20130415.bam	HG02760	GWD	BGI
HG02763.mapped.ILLUMINA.bwa.GWD.exome.20130415.bam	HG02763	GWD	WUGSC
HG02771.mapped.ILLUMINA.bwa.GWD.exome.20130415.bam	HG02771	GWD	BI
HG02774.mapped.ILLUMINA.bwa.PJL.exome.20130415.bam	HG02774	PJL	BGI
HG02775.mapped.ILLUMINA.bwa.PJL.exome.20130415.bam	HG02775	PJL	BGI
HG02778.mapped.ILLUMINA.bwa.PJL.exome.20130415.bam	HG02778	PJL	WUGSC
HG02780.mapped.ILLUMINA.bwa.PJL.exome.20130415.bam	HG02780	PJL	WUGSC
HG02786.mapped.ILLUMINA.bwa.PJL.exome.20130415.bam	HG02786	PJL	BI
HG02792.mapped.ILLUMINA.bwa.PJL.exome.20130415.bam	HG02792	PJL	BGI
HG02793.mapped.ILLUMINA.bwa.PJL.exome.20130415.bam	HG02793	PJL	BGI

HG02798.mapped.ILLUMINA.bwa.GWD.exome.20130415.bam	HG02798	GWD	BI
HG02799.mapped.ILLUMINA.bwa.GWD.exome.20130415.bam	HG02799	GWD	BI
HG02804.mapped.ILLUMINA.bwa.GWD.exome.20130415.bam	HG02804	GWD	BI
HG02805.mapped.ILLUMINA.bwa.GWD.exome.20130415.bam	HG02805	GWD	BI
HG02808.mapped.ILLUMINA.bwa.GWD.exome.20130415.bam	HG02808	GWD	BI
HG02810.mapped.ILLUMINA.bwa.GWD.exome.20130415.bam	HG02810	GWD	BI
HG02813.mapped.ILLUMINA.bwa.GWD.exome.20130415.bam	HG02813	GWD	BGI
HG02814.mapped.ILLUMINA.bwa.GWD.exome.20130415.bam	HG02814	GWD	BGI
HG02816.mapped.ILLUMINA.bwa.GWD.exome.20130415.bam	HG02816	GWD	BGI
HG02817.mapped.ILLUMINA.bwa.GWD.exome.20130415.bam	HG02817	GWD	BGI
HG02819.mapped.ILLUMINA.bwa.GWD.exome.20130415.bam	HG02819	GWD	BI
HG02820.mapped.ILLUMINA.bwa.GWD.exome.20130415.bam	HG02820	GWD	BI
HG02836.mapped.ILLUMINA.bwa.GWD.exome.20130415.bam	HG02836	GWD	BGI
HG02837.mapped.ILLUMINA.bwa.GWD.exome.20130415.bam	HG02837	GWD	BGI
HG02839.mapped.ILLUMINA.bwa.GWD.exome.20130415.bam	HG02839	GWD	BGI
HG02840.mapped.ILLUMINA.bwa.GWD.exome.20130415.bam	HG02840	GWD	BGI
HG02851.mapped.ILLUMINA.bwa.GWD.exome.20130415.bam	HG02851	GWD	BGI
HG02852.mapped.ILLUMINA.bwa.GWD.exome.20130415.bam	HG02852	GWD	BGI
HG02854.mapped.ILLUMINA.bwa.GWD.exome.20130415.bam	HG02854	GWD	BI
HG02855.mapped.ILLUMINA.bwa.GWD.exome.20130415.bam	HG02855	GWD	BI
HG02860.mapped.ILLUMINA.bwa.GWD.exome.20130415.bam	HG02860	GWD	BGI
HG02861.mapped.ILLUMINA.bwa.GWD.exome.20130415.bam	HG02861	GWD	BGI
HG02870.mapped.ILLUMINA.bwa.GWD.exome.20130415.bam	HG02870	GWD	WUGSC
HG02878.mapped.ILLUMINA.bwa.GWD.exome.20130415.bam	HG02878	GWD	BGI
HG02879.mapped.ILLUMINA.bwa.GWD.exome.20130415.bam	HG02879	GWD	BGI
HG02881.mapped.ILLUMINA.bwa.GWD.exome.20130415.bam	HG02881	GWD	BGI
HG02882.mapped.ILLUMINA.bwa.GWD.exome.20130415.bam	HG02882	GWD	BGI
HG02884.mapped.ILLUMINA.bwa.GWD.exome.20130415.bam	HG02884	GWD	BI
HG02885.mapped.ILLUMINA.bwa.GWD.exome.20130415.bam	HG02885	GWD	BI
HG02887.mapped.ILLUMINA.bwa.GWD.exome.20130415.bam	HG02887	GWD	BGI

HG02888.mapped.ILLUMINA.bwa.GWD.exome.20130415.bam	HG02888	GWD	BGI
HG02890.mapped.ILLUMINA.bwa.GWD.exome.20130415.bam	HG02890	GWD	BI
HG02891.mapped.ILLUMINA.bwa.GWD.exome.20130415.bam	HG02891	GWD	BI
HG02895.mapped.ILLUMINA.bwa.GWD.exome.20130415.bam	HG02895	GWD	BGI
HG02896.mapped.ILLUMINA.bwa.GWD.exome.20130415.bam	HG02896	GWD	BGI
HG02938.mapped.ILLUMINA.bwa.ESN.exome.20130415.bam	HG02938	ESN	BGI
HG02941.mapped.ILLUMINA.bwa.ESN.exome.20130415.bam	HG02941	ESN	BGI
HG02943.mapped.ILLUMINA.bwa.ESN.exome.20130415.bam	HG02943	ESN	BI
HG02946.mapped.ILLUMINA.bwa.ESN.exome.20130415.bam	HG02946	ESN	BGI
HG02947.mapped.ILLUMINA.bwa.ESN.exome.20130415.bam	HG02947	ESN	BGI
HG02952.mapped.ILLUMINA.bwa.ESN.exome.20130415.bam	HG02952	ESN	BGI
HG02953.mapped.ILLUMINA.bwa.ESN.exome.20130415.bam	HG02953	ESN	BGI
HG02968.mapped.ILLUMINA.bwa.ESN.exome.20130415.bam	HG02968	ESN	BGI
HG02970.mapped.ILLUMINA.bwa.ESN.exome.20130415.bam	HG02970	ESN	BGI
HG02971.mapped.ILLUMINA.bwa.ESN.exome.20130415.bam	HG02971	ESN	BGI
HG02976.mapped.ILLUMINA.bwa.ESN.exome.20130415.bam	HG02976	ESN	BGI
HG02977.mapped.ILLUMINA.bwa.ESN.exome.20130415.bam	HG02977	ESN	BGI
HG02979.mapped.ILLUMINA.bwa.ESN.exome.20130415.bam	HG02979	ESN	BGI
HG02981.mapped.ILLUMINA.bwa.ESN.exome.20130415.bam	HG02981	ESN	BGI
HG02982.mapped.ILLUMINA.bwa.GWD.exome.20130415.bam	HG02982	GWD	BGI
HG02983.mapped.ILLUMINA.bwa.GWD.exome.20130415.bam	HG02983	GWD	BGI
HG03006.mapped.ILLUMINA.bwa.BEB.exome.20130415.bam	HG03006	BEB	BI
HG03007.mapped.ILLUMINA.bwa.BEB.exome.20130415.bam	HG03007	BEB	BI
HG03015.mapped.ILLUMINA.bwa.PJL.exome.20130415.bam	HG03015	PJL	BGI
HG03016.mapped.ILLUMINA.bwa.PJL.exome.20130415.bam	HG03016	PJL	BGI
HG03018.mapped.ILLUMINA.bwa.PJL.exome.20130415.bam	HG03018	PJL	WUGSC
HG03019.mapped.ILLUMINA.bwa.PJL.exome.20130415.bam	HG03019	PJL	WUGSC
HG03021.mapped.ILLUMINA.bwa.PJL.exome.20130415.bam	HG03021	PJL	WUGSC
HG03022.mapped.ILLUMINA.bwa.PJL.exome.20130415.bam	HG03022	PJL	WUGSC
HG03024.mapped.ILLUMINA.bwa.GWD.exome.20130415.bam	HG03024	GWD	BI

HG03025.mapped.ILLUMINA.bwa.GWD.exome.20130415.bam	HG03025	GWD	BI
HG03027.mapped.ILLUMINA.bwa.GWD.exome.20130415.bam	HG03027	GWD	BI
HG03028.mapped.ILLUMINA.bwa.GWD.exome.20130415.bam	HG03028	GWD	BI
HG03039.mapped.ILLUMINA.bwa.GWD.exome.20130415.bam	HG03039	GWD	BI
HG03040.mapped.ILLUMINA.bwa.GWD.exome.20130415.bam	HG03040	GWD	BI
HG03045.mapped.ILLUMINA.bwa.GWD.exome.20130415.bam	HG03045	GWD	BI
HG03049.mapped.ILLUMINA.bwa.GWD.exome.20130415.bam	HG03049	GWD	BI
HG03054.mapped.ILLUMINA.bwa.MSL.exome.20130415.bam	HG03054	MSL	BGI
HG03055.mapped.ILLUMINA.bwa.MSL.exome.20130415.bam	HG03055	MSL	BGI
HG03060.mapped.ILLUMINA.bwa.MSL.exome.20130415.bam	HG03060	MSL	BGI
HG03061.mapped.ILLUMINA.bwa.MSL.exome.20130415.bam	HG03061	MSL	BGI
HG03063.mapped.ILLUMINA.bwa.MSL.exome.20130415.bam	HG03063	MSL	BGI
HG03064.mapped.ILLUMINA.bwa.MSL.exome.20130415.bam	HG03064	MSL	BGI
HG03066.mapped.ILLUMINA.bwa.MSL.exome.20130415.bam	HG03066	MSL	BI
HG03069.mapped.ILLUMINA.bwa.MSL.exome.20130415.bam	HG03069	MSL	BI
HG03072.mapped.ILLUMINA.bwa.MSL.exome.20130415.bam	HG03072	MSL	BI
HG03073.mapped.ILLUMINA.bwa.MSL.exome.20130415.bam	HG03073	MSL	BGI
HG03074.mapped.ILLUMINA.bwa.MSL.exome.20130415.bam	HG03074	MSL	BGI
HG03077.mapped.ILLUMINA.bwa.MSL.exome.20130415.bam	HG03077	MSL	BGI
HG03079.mapped.ILLUMINA.bwa.MSL.exome.20130415.bam	HG03079	MSL	BGI
HG03081.mapped.ILLUMINA.bwa.MSL.exome.20130415.bam	HG03081	MSL	BGI
HG03082.mapped.ILLUMINA.bwa.MSL.exome.20130415.bam	HG03082	MSL	BGI
HG03086.mapped.ILLUMINA.bwa.MSL.exome.20130415.bam	HG03086	MSL	BGI
HG03088.mapped.ILLUMINA.bwa.MSL.exome.20130415.bam	HG03088	MSL	BGI
HG03091.mapped.ILLUMINA.bwa.MSL.exome.20130415.bam	HG03091	MSL	BGI
HG03095.mapped.ILLUMINA.bwa.MSL.exome.20130415.bam	HG03095	MSL	BGI
HG03103.mapped.ILLUMINA.bwa.ESN.exome.20130415.bam	HG03103	ESN	BGI
HG03105.mapped.ILLUMINA.bwa.ESN.exome.20130415.bam	HG03105	ESN	BGI
HG03111.mapped.ILLUMINA.bwa.ESN.exome.20130415.bam	HG03111	ESN	BGI
HG03112.mapped.ILLUMINA.bwa.ESN.exome.20130415.bam	HG03112	ESN	BGI

HG03117.mapped.ILLUMINA.bwa.ESN.exome.20130415.bam	HG03117	ESN	BI
HG03118.mapped.ILLUMINA.bwa.ESN.exome.20130415.bam	HG03118	ESN	BI
HG03126.mapped.ILLUMINA.bwa.ESN.exome.20130415.bam	HG03126	ESN	BGI
HG03127.mapped.ILLUMINA.bwa.ESN.exome.20130415.bam	HG03127	ESN	BGI
HG03129.mapped.ILLUMINA.bwa.ESN.exome.20130415.bam	HG03129	ESN	BI
HG03130.mapped.ILLUMINA.bwa.ESN.exome.20130415.bam	HG03130	ESN	BI
HG03132.mapped.ILLUMINA.bwa.ESN.exome.20130415.bam	HG03132	ESN	BI
HG03136.mapped.ILLUMINA.bwa.ESN.exome.20130415.bam	HG03136	ESN	BI
HG03139.mapped.ILLUMINA.bwa.ESN.exome.20130415.bam	HG03139	ESN	BGI
HG03157.mapped.ILLUMINA.bwa.ESN.exome.20130415.bam	HG03157	ESN	BGI
HG03159.mapped.ILLUMINA.bwa.ESN.exome.20130415.bam	HG03159	ESN	BI
HG03162.mapped.ILLUMINA.bwa.ESN.exome.20130415.bam	HG03162	ESN	BI
HG03163.mapped.ILLUMINA.bwa.ESN.exome.20130415.bam	HG03163	ESN	BI
HG03166.mapped.ILLUMINA.bwa.ESN.exome.20130415.bam	HG03166	ESN	BGI
HG03168.mapped.ILLUMINA.bwa.ESN.exome.20130415.bam	HG03168	ESN	BI
HG03169.mapped.ILLUMINA.bwa.ESN.exome.20130415.bam	HG03169	ESN	BI
HG03172.mapped.ILLUMINA.bwa.ESN.exome.20130415.bam	HG03172	ESN	BI
HG03175.mapped.ILLUMINA.bwa.ESN.exome.20130415.bam	HG03175	ESN	BGI
HG03189.mapped.ILLUMINA.bwa.ESN.exome.20130415.bam	HG03189	ESN	BGI
HG03190.mapped.ILLUMINA.bwa.ESN.exome.20130415.bam	HG03190	ESN	BGI
HG03193.mapped.ILLUMINA.bwa.ESN.exome.20130415.bam	HG03193	ESN	BGI
HG03195.mapped.ILLUMINA.bwa.ESN.exome.20130415.bam	HG03195	ESN	BI
HG03196.mapped.ILLUMINA.bwa.ESN.exome.20130415.bam	HG03196	ESN	BI
HG03198.mapped.ILLUMINA.bwa.ESN.exome.20130415.bam	HG03198	ESN	BI
HG03199.mapped.ILLUMINA.bwa.ESN.exome.20130415.bam	HG03199	ESN	BI
HG03202.mapped.ILLUMINA.bwa.ESN.exome.20130415.bam	HG03202	ESN	BGI
HG03209.mapped.ILLUMINA.bwa.MSL.exome.20130415.bam	HG03209	MSL	BGI
HG03212.mapped.ILLUMINA.bwa.MSL.exome.20130415.bam	HG03212	MSL	BGI
HG03224.mapped.ILLUMINA.bwa.MSL.exome.20130415.bam	HG03224	MSL	BGI
HG03225.mapped.ILLUMINA.bwa.MSL.exome.20130415.bam	HG03225	MSL	BGI

HG03228.mapped.ILLUMINA.bwa.PJL.exome.20130415.bam	HG03228	PJL	BGI
HG03229.mapped.ILLUMINA.bwa.PJL.exome.20130415.bam	HG03229	PJL	BGI
HG03234.mapped.ILLUMINA.bwa.PJL.exome.20130415.bam	HG03234	PJL	BGI
HG03235.mapped.ILLUMINA.bwa.PJL.exome.20130415.bam	HG03235	PJL	BGI
HG03237.mapped.ILLUMINA.bwa.PJL.exome.20130415.bam	HG03237	PJL	BGI
HG03238.mapped.ILLUMINA.bwa.PJL.exome.20130415.bam	HG03238	PJL	BGI
HG03240.mapped.ILLUMINA.bwa.GWD.exome.20130415.bam	HG03240	GWD	BI
HG03241.mapped.ILLUMINA.bwa.GWD.exome.20130415.bam	HG03241	GWD	BI
HG03246.mapped.ILLUMINA.bwa.GWD.exome.20130415.bam	HG03246	GWD	BI
HG03247.mapped.ILLUMINA.bwa.GWD.exome.20130415.bam	HG03247	GWD	BI
HG03258.mapped.ILLUMINA.bwa.GWD.exome.20130415.bam	HG03258	GWD	BI
HG03259.mapped.ILLUMINA.bwa.GWD.exome.20130415.bam	HG03259	GWD	BI
HG03265.mapped.ILLUMINA.bwa.ESN.exome.20130415.bam	HG03265	ESN	BGI
HG03267.mapped.ILLUMINA.bwa.ESN.exome.20130415.bam	HG03267	ESN	BGI
HG03268.mapped.ILLUMINA.bwa.ESN.exome.20130415.bam	HG03268	ESN	BGI
HG03270.mapped.ILLUMINA.bwa.ESN.exome.20130415.bam	HG03270	ESN	BGI
HG03271.mapped.ILLUMINA.bwa.ESN.exome.20130415.bam	HG03271	ESN	BGI
HG03279.mapped.ILLUMINA.bwa.ESN.exome.20130415.bam	HG03279	ESN	BGI
HG03280.mapped.ILLUMINA.bwa.ESN.exome.20130415.bam	HG03280	ESN	BGI
HG03291.mapped.ILLUMINA.bwa.ESN.exome.20130415.bam	HG03291	ESN	BGI
HG03294.mapped.ILLUMINA.bwa.ESN.exome.20130415.bam	HG03294	ESN	BGI
HG03295.mapped.ILLUMINA.bwa.ESN.exome.20130415.bam	HG03295	ESN	BGI
HG03297.mapped.ILLUMINA.bwa.ESN.exome.20130415.bam	HG03297	ESN	BGI
HG03298.mapped.ILLUMINA.bwa.ESN.exome.20130415.bam	HG03298	ESN	BGI
HG03300.mapped.ILLUMINA.bwa.ESN.exome.20130415.bam	HG03300	ESN	BGI
HG03301.mapped.ILLUMINA.bwa.ESN.exome.20130415.bam	HG03301	ESN	BGI
HG03303.mapped.ILLUMINA.bwa.ESN.exome.20130415.bam	HG03303	ESN	BGI
HG03304.mapped.ILLUMINA.bwa.ESN.exome.20130415.bam	HG03304	ESN	BGI
HG03311.mapped.ILLUMINA.bwa.ESN.exome.20130415.bam	HG03311	ESN	WUGSC
HG03313.mapped.ILLUMINA.bwa.ESN.exome.20130415.bam	HG03313	ESN	WUGSC

HG03342.mapped.ILLUMINA.bwa.ESN.exome.20130415.bam	HG03342	ESN	BI
HG03343.mapped.ILLUMINA.bwa.ESN.exome.20130415.bam	HG03343	ESN	BI
HG03351.mapped.ILLUMINA.bwa.ESN.exome.20130415.bam	HG03351	ESN	WUGSC
HG03352.mapped.ILLUMINA.bwa.ESN.exome.20130415.bam	HG03352	ESN	WUGSC
HG03354.mapped.ILLUMINA.bwa.ESN.exome.20130415.bam	HG03354	ESN	BGI
HG03363.mapped.ILLUMINA.bwa.ESN.exome.20130415.bam	HG03363	ESN	WUGSC
HG03366.mapped.ILLUMINA.bwa.ESN.exome.20130415.bam	HG03366	ESN	BGI
HG03367.mapped.ILLUMINA.bwa.ESN.exome.20130415.bam	HG03367	ESN	BGI
HG03369.mapped.ILLUMINA.bwa.ESN.exome.20130415.bam	HG03369	ESN	WUGSC
HG03370.mapped.ILLUMINA.bwa.ESN.exome.20130415.bam	HG03370	ESN	WUGSC
HG03372.mapped.ILLUMINA.bwa.ESN.exome.20130415.bam	HG03372	ESN	WUGSC
HG03376.mapped.ILLUMINA.bwa.MSL.exome.20130415.bam	HG03376	MSL	BGI
HG03378.mapped.ILLUMINA.bwa.MSL.exome.20130415.bam	HG03378	MSL	BGI
HG03380.mapped.ILLUMINA.bwa.MSL.exome.20130415.bam	HG03380	MSL	BGI
HG03382.mapped.ILLUMINA.bwa.MSL.exome.20130415.bam	HG03382	MSL	BGI
HG03385.mapped.ILLUMINA.bwa.MSL.exome.20130415.bam	HG03385	MSL	BGI
HG03388.mapped.ILLUMINA.bwa.MSL.exome.20130415.bam	HG03388	MSL	BGI
HG03391.mapped.ILLUMINA.bwa.MSL.exome.20130415.bam	HG03391	MSL	BI
HG03394.mapped.ILLUMINA.bwa.MSL.exome.20130415.bam	HG03394	MSL	WUGSC
HG03401.mapped.ILLUMINA.bwa.MSL.exome.20130415.bam	HG03401	MSL	BGI
HG03410.mapped.ILLUMINA.bwa.MSL.exome.20130415.bam	HG03410	MSL	BGI
HG03419.mapped.ILLUMINA.bwa.MSL.exome.20130415.bam	HG03419	MSL	BGI
HG03428.mapped.ILLUMINA.bwa.MSL.exome.20130415.bam	HG03428	MSL	BGI
HG03432.mapped.ILLUMINA.bwa.MSL.exome.20130415.bam	HG03432	MSL	WUGSC
HG03433.mapped.ILLUMINA.bwa.MSL.exome.20130415.bam	HG03433	MSL	BGI
HG03436.mapped.ILLUMINA.bwa.MSL.exome.20130415.bam	HG03436	MSL	BI
HG03437.mapped.ILLUMINA.bwa.MSL.exome.20130415.bam	HG03437	MSL	BI
HG03439.mapped.ILLUMINA.bwa.MSL.exome.20130415.bam	HG03439	MSL	BGI
HG03442.mapped.ILLUMINA.bwa.MSL.exome.20130415.bam	HG03442	MSL	BGI
HG03445.mapped.ILLUMINA.bwa.MSL.exome.20130415.bam	HG03445	MSL	BGI

HG03446.mapped.ILLUMINA.bwa.MSL.exome.20130415.bam	HG03446	MSL	BGI
HG03449.mapped.ILLUMINA.bwa.MSL.exome.20130415.bam	HG03449	MSL	BGI
HG03452.mapped.ILLUMINA.bwa.MSL.exome.20130415.bam	HG03452	MSL	BI
HG03455.mapped.ILLUMINA.bwa.MSL.exome.20130415.bam	HG03455	MSL	BI
HG03457.mapped.ILLUMINA.bwa.MSL.exome.20130415.bam	HG03457	MSL	WUGSC
HG03458.mapped.ILLUMINA.bwa.MSL.exome.20130415.bam	HG03458	MSL	BI
HG03460.mapped.ILLUMINA.bwa.MSL.exome.20130415.bam	HG03460	MSL	BGI
HG03461.mapped.ILLUMINA.bwa.MSL.exome.20130415.bam	HG03461	MSL	BGI
HG03470.mapped.ILLUMINA.bwa.MSL.exome.20130415.bam	HG03470	MSL	BGI
HG03473.mapped.ILLUMINA.bwa.MSL.exome.20130415.bam	HG03473	MSL	BGI
HG03476.mapped.ILLUMINA.bwa.MSL.exome.20130415.bam	HG03476	MSL	BI
HG03478.mapped.ILLUMINA.bwa.MSL.exome.20130415.bam	HG03478	MSL	BGI
HG03479.mapped.ILLUMINA.bwa.MSL.exome.20130415.bam	HG03479	MSL	BGI
HG03484.mapped.ILLUMINA.bwa.MSL.exome.20130415.bam	HG03484	MSL	BI
HG03485.mapped.ILLUMINA.bwa.MSL.exome.20130415.bam	HG03485	MSL	BI
HG03490.mapped.ILLUMINA.bwa.PJL.exome.20130415.bam	HG03490	PJL	WUGSC
HG03491.mapped.ILLUMINA.bwa.PJL.exome.20130415.bam	HG03491	PJL	WUGSC
HG03499.mapped.ILLUMINA.bwa.ESN.exome.20130415.bam	HG03499	ESN	WUGSC
HG03511.mapped.ILLUMINA.bwa.ESN.exome.20130415.bam	HG03511	ESN	WUGSC
HG03514.mapped.ILLUMINA.bwa.ESN.exome.20130415.bam	HG03514	ESN	BGI
HG03515.mapped.ILLUMINA.bwa.ESN.exome.20130415.bam	HG03515	ESN	BGI
HG03517.mapped.ILLUMINA.bwa.ESN.exome.20130415.bam	HG03517	ESN	WUGSC
HG03518.mapped.ILLUMINA.bwa.ESN.exome.20130415.bam	HG03518	ESN	WUGSC
HG03520.mapped.ILLUMINA.bwa.ESN.exome.20130415.bam	HG03520	ESN	BI
HG03521.mapped.ILLUMINA.bwa.ESN.exome.20130415.bam	HG03521	ESN	BI
HG03538.mapped.ILLUMINA.bwa.GWD.exome.20130415.bam	HG03538	GWD	BI
HG03539.mapped.ILLUMINA.bwa.GWD.exome.20130415.bam	HG03539	GWD	BI
HG03547.mapped.ILLUMINA.bwa.MSL.exome.20130415.bam	HG03547	MSL	BI
HG03548.mapped.ILLUMINA.bwa.MSL.exome.20130415.bam	HG03548	MSL	BI
HG03556.mapped.ILLUMINA.bwa.MSL.exome.20130415.bam	HG03556	MSL	BI

HG03557.mapped.ILLUMINA.bwa.MSL.exome.20130415.bam	HG03557	MSL	BI
HG03558.mapped.ILLUMINA.bwa.MSL.exome.20130415.bam	HG03558	MSL	WUGSC
HG03559.mapped.ILLUMINA.bwa.MSL.exome.20130415.bam	HG03559	MSL	WUGSC
HG03565.mapped.ILLUMINA.bwa.MSL.exome.20130415.bam	HG03565	MSL	WUGSC
HG03567.mapped.ILLUMINA.bwa.MSL.exome.20130415.bam	HG03567	MSL	WUGSC
HG03571.mapped.ILLUMINA.bwa.MSL.exome.20130415.bam	HG03571	MSL	BGI
HG03572.mapped.ILLUMINA.bwa.MSL.exome.20130415.bam	HG03572	MSL	BI
HG03575.mapped.ILLUMINA.bwa.MSL.exome.20130415.bam	HG03575	MSL	BI
HG03577.mapped.ILLUMINA.bwa.MSL.exome.20130415.bam	HG03577	MSL	BI
HG03578.mapped.ILLUMINA.bwa.MSL.exome.20130415.bam	HG03578	MSL	BI
HG03583.mapped.ILLUMINA.bwa.MSL.exome.20130415.bam	HG03583	MSL	BI
HG03594.mapped.ILLUMINA.bwa.BEB.exome.20130415.bam	HG03594	BEB	BGI
HG03595.mapped.ILLUMINA.bwa.BEB.exome.20130415.bam	HG03595	BEB	BGI
HG03598.mapped.ILLUMINA.bwa.BEB.exome.20130415.bam	HG03598	BEB	WUGSC
HG03600.mapped.ILLUMINA.bwa.BEB.exome.20130415.bam	HG03600	BEB	BGI
HG03603.mapped.ILLUMINA.bwa.BEB.exome.20130415.bam	HG03603	BEB	BGI
HG03604.mapped.ILLUMINA.bwa.BEB.exome.20130415.bam	HG03604	BEB	BGI
HG03607.mapped.ILLUMINA.bwa.BEB.exome.20130415.bam	HG03607	BEB	BGI
HG03611.mapped.ILLUMINA.bwa.BEB.exome.20130415.bam	HG03611	BEB	BGI
HG03615.mapped.ILLUMINA.bwa.BEB.exome.20130415.bam	HG03615	BEB	BI
HG03616.mapped.ILLUMINA.bwa.BEB.exome.20130415.bam	HG03616	BEB	BI
HG03619.mapped.ILLUMINA.bwa.PJL.exome.20130415.bam	HG03619	PJL	BGI
HG03624.mapped.ILLUMINA.bwa.PJL.exome.20130415.bam	HG03624	PJL	WUGSC
HG03625.mapped.ILLUMINA.bwa.PJL.exome.20130415.bam	HG03625	PJL	WUGSC
HG03629.mapped.ILLUMINA.bwa.PJL.exome.20130415.bam	HG03629	PJL	WUGSC
HG03631.mapped.ILLUMINA.bwa.PJL.exome.20130415.bam	HG03631	PJL	WUGSC
HG03634.mapped.ILLUMINA.bwa.PJL.exome.20130415.bam	HG03634	PJL	BGI
HG03636.mapped.ILLUMINA.bwa.PJL.exome.20130415.bam	HG03636	PJL	WUGSC
HG03640.mapped.ILLUMINA.bwa.PJL.exome.20130415.bam	HG03640	PJL	WUGSC
HG03642.mapped.ILLUMINA.bwa.STU.exome.20130415.bam	HG03642	STU	BI

HG03643.mapped.ILLUMINA.bwa.STU.exome.20130415.bam	HG03643	STU	BI
HG03644.mapped.ILLUMINA.bwa.STU.exome.20130415.bam	HG03644	STU	BI
HG03645.mapped.ILLUMINA.bwa.STU.exome.20130415.bam	HG03645	STU	BGI
HG03646.mapped.ILLUMINA.bwa.STU.exome.20130415.bam	HG03646	STU	BGI
HG03649.mapped.ILLUMINA.bwa.PJL.exome.20130415.bam	HG03649	PJL	WUGSC
HG03652.mapped.ILLUMINA.bwa.PJL.exome.20130415.bam	HG03652	PJL	WUGSC
HG03653.mapped.ILLUMINA.bwa.PJL.exome.20130415.bam	HG03653	PJL	WUGSC
HG03667.mapped.ILLUMINA.bwa.PJL.exome.20130415.bam	HG03667	PJL	BGI
HG03668.mapped.ILLUMINA.bwa.PJL.exome.20130415.bam	HG03668	PJL	BGI
HG03672.mapped.ILLUMINA.bwa.STU.exome.20130415.bam	HG03672	STU	BGI
HG03680.mapped.ILLUMINA.bwa.STU.exome.20130415.bam	HG03680	STU	BI
HG03681.mapped.ILLUMINA.bwa.STU.exome.20130415.bam	HG03681	STU	BI
HG03684.mapped.ILLUMINA.bwa.STU.exome.20130415.bam	HG03684	STU	BI
HG03685.mapped.ILLUMINA.bwa.STU.exome.20130415.bam	HG03685	STU	BI
HG03686.mapped.ILLUMINA.bwa.STU.exome.20130415.bam	HG03686	STU	BGI
HG03687.mapped.ILLUMINA.bwa.STU.exome.20130415.bam	HG03687	STU	BGI
HG03689.mapped.ILLUMINA.bwa.STU.exome.20130415.bam	HG03689	STU	BGI
HG03690.mapped.ILLUMINA.bwa.STU.exome.20130415.bam	HG03690	STU	BGI
HG03691.mapped.ILLUMINA.bwa.STU.exome.20130415.bam	HG03691	STU	BGI
HG03693.mapped.ILLUMINA.bwa.STU.exome.20130415.bam	HG03693	STU	BI
HG03694.mapped.ILLUMINA.bwa.STU.exome.20130415.bam	HG03694	STU	BGI
HG03695.mapped.ILLUMINA.bwa.STU.exome.20130415.bam	HG03695	STU	BGI
HG03696.mapped.ILLUMINA.bwa.STU.exome.20130415.bam	HG03696	STU	BGI
HG03697.mapped.ILLUMINA.bwa.STU.exome.20130415.bam	HG03697	STU	BGI
HG03698.mapped.ILLUMINA.bwa.STU.exome.20130415.bam	HG03698	STU	BGI
HG03702.mapped.ILLUMINA.bwa.PJL.exome.20130415.bam	HG03702	PJL	BGI
HG03703.mapped.ILLUMINA.bwa.PJL.exome.20130415.bam	HG03703	PJL	BGI
HG03705.mapped.ILLUMINA.bwa.PJL.exome.20130415.bam	HG03705	PJL	WUGSC
HG03706.mapped.ILLUMINA.bwa.PJL.exome.20130415.bam	HG03706	PJL	WUGSC
HG03708.mapped.ILLUMINA.bwa.PJL.exome.20130415.bam	HG03708	PJL	BGI

HG03709.mapped.ILLUMINA.bwa.PJL.exome.20130415.bam	HG03709	PJL	BGI
HG03711.mapped.ILLUMINA.bwa.STU.exome.20130415.bam	HG03711	STU	BGI
HG03713.mapped.ILLUMINA.bwa.ITU.exome.20130415.bam	HG03713	ITU	BI
HG03714.mapped.ILLUMINA.bwa.ITU.exome.20130415.bam	HG03714	ITU	BI
HG03715.mapped.ILLUMINA.bwa.ITU.exome.20130415.bam	HG03715	ITU	BI
HG03716.mapped.ILLUMINA.bwa.ITU.exome.20130415.bam	HG03716	ITU	BI
HG03718.mapped.ILLUMINA.bwa.ITU.exome.20130415.bam	HG03718	ITU	WUGSC
HG03720.mapped.ILLUMINA.bwa.ITU.exome.20130415.bam	HG03720	ITU	BI
HG03722.mapped.ILLUMINA.bwa.ITU.exome.20130415.bam	HG03722	ITU	BI
HG03727.mapped.ILLUMINA.bwa.ITU.exome.20130415.bam	HG03727	ITU	BI
HG03729.mapped.ILLUMINA.bwa.ITU.exome.20130415.bam	HG03729	ITU	BI
HG03730.mapped.ILLUMINA.bwa.ITU.exome.20130415.bam	HG03730	ITU	BI
HG03731.mapped.ILLUMINA.bwa.ITU.exome.20130415.bam	HG03731	ITU	BI
HG03733.mapped.ILLUMINA.bwa.STU.exome.20130415.bam	HG03733	STU	BGI
HG03736.mapped.ILLUMINA.bwa.STU.exome.20130415.bam	HG03736	STU	BGI
HG03738.mapped.ILLUMINA.bwa.STU.exome.20130415.bam	HG03738	STU	BGI
HG03740.mapped.ILLUMINA.bwa.STU.exome.20130415.bam	HG03740	STU	BI
HG03742.mapped.ILLUMINA.bwa.ITU.exome.20130415.bam	HG03742	ITU	BGI
HG03743.mapped.ILLUMINA.bwa.STU.exome.20130415.bam	HG03743	STU	BI
HG03744.mapped.ILLUMINA.bwa.STU.exome.20130415.bam	HG03744	STU	BGI
HG03745.mapped.ILLUMINA.bwa.STU.exome.20130415.bam	HG03745	STU	BGI
HG03746.mapped.ILLUMINA.bwa.STU.exome.20130415.bam	HG03746	STU	BI
HG03750.mapped.ILLUMINA.bwa.STU.exome.20130415.bam	HG03750	STU	BI
HG03752.mapped.ILLUMINA.bwa.STU.exome.20130415.bam	HG03752	STU	BGI
HG03753.mapped.ILLUMINA.bwa.STU.exome.20130415.bam	HG03753	STU	BGI
HG03754.mapped.ILLUMINA.bwa.STU.exome.20130415.bam	HG03754	STU	BI
HG03755.mapped.ILLUMINA.bwa.STU.exome.20130415.bam	HG03755	STU	BI
HG03756.mapped.ILLUMINA.bwa.STU.exome.20130415.bam	HG03756	STU	BI
HG03760.mapped.ILLUMINA.bwa.STU.exome.20130415.bam	HG03760	STU	BGI
HG03762.mapped.ILLUMINA.bwa.PJL.exome.20130415.bam	HG03762	PJL	WUGSC

HG03765.mapped.ILLUMINA.bwa.PJL.exome.20130415.bam	HG03765	PJL	WUGSC
HG03767.mapped.ILLUMINA.bwa.PJL.exome.20130415.bam	HG03767	PJL	WUGSC
HG03770.mapped.ILLUMINA.bwa.ITU.exome.20130415.bam	HG03770	ITU	BI
HG03771.mapped.ILLUMINA.bwa.ITU.exome.20130415.bam	HG03771	ITU	BI
HG03772.mapped.ILLUMINA.bwa.ITU.exome.20130415.bam	HG03772	ITU	BI
HG03773.mapped.ILLUMINA.bwa.ITU.exome.20130415.bam	HG03773	ITU	BI
HG03774.mapped.ILLUMINA.bwa.ITU.exome.20130415.bam	HG03774	ITU	WUGSC
HG03775.mapped.ILLUMINA.bwa.ITU.exome.20130415.bam	HG03775	ITU	BI
HG03777.mapped.ILLUMINA.bwa.ITU.exome.20130415.bam	HG03777	ITU	BGI
HG03778.mapped.ILLUMINA.bwa.ITU.exome.20130415.bam	HG03778	ITU	BGI
HG03779.mapped.ILLUMINA.bwa.ITU.exome.20130415.bam	HG03779	ITU	BGI
HG03780.mapped.ILLUMINA.bwa.ITU.exome.20130415.bam	HG03780	ITU	WUGSC
HG03781.mapped.ILLUMINA.bwa.ITU.exome.20130415.bam	HG03781	ITU	BI
HG03782.mapped.ILLUMINA.bwa.ITU.exome.20130415.bam	HG03782	ITU	BGI
HG03784.mapped.ILLUMINA.bwa.ITU.exome.20130415.bam	HG03784	ITU	BI
HG03785.mapped.ILLUMINA.bwa.ITU.exome.20130415.bam	HG03785	ITU	BGI
HG03786.mapped.ILLUMINA.bwa.ITU.exome.20130415.bam	HG03786	ITU	BI
HG03787.mapped.ILLUMINA.bwa.ITU.exome.20130415.bam	HG03787	ITU	BI
HG03788.mapped.ILLUMINA.bwa.ITU.exome.20130415.bam	HG03788	ITU	BI
HG03789.mapped.ILLUMINA.bwa.ITU.exome.20130415.bam	HG03789	ITU	BI
HG03790.mapped.ILLUMINA.bwa.ITU.exome.20130415.bam	HG03790	ITU	BGI
HG03792.mapped.ILLUMINA.bwa.ITU.exome.20130415.bam	HG03792	ITU	BGI
HG03793.mapped.ILLUMINA.bwa.BEB.exome.20130415.bam	HG03793	BEB	BI
HG03796.mapped.ILLUMINA.bwa.BEB.exome.20130415.bam	HG03796	BEB	BI
HG03800.mapped.ILLUMINA.bwa.BEB.exome.20130415.bam	HG03800	BEB	BI
HG03802.mapped.ILLUMINA.bwa.BEB.exome.20130415.bam	HG03802	BEB	BI
HG03803.mapped.ILLUMINA.bwa.BEB.exome.20130415.bam	HG03803	BEB	BI
HG03805.mapped.ILLUMINA.bwa.BEB.exome.20130415.bam	HG03805	BEB	BGI
HG03808.mapped.ILLUMINA.bwa.BEB.exome.20130415.bam	HG03808	BEB	BGI
HG03809.mapped.ILLUMINA.bwa.BEB.exome.20130415.bam	HG03809	BEB	BGI

HG03814.mapped.ILLUMINA.bwa.BEB.exome.20130415.bam	HG03814	BEB	BGI
HG03815.mapped.ILLUMINA.bwa.BEB.exome.20130415.bam	HG03815	BEB	BI
HG03821.mapped.ILLUMINA.bwa.BEB.exome.20130415.bam	HG03821	BEB	WUGSC
HG03823.mapped.ILLUMINA.bwa.BEB.exome.20130415.bam	HG03823	BEB	BGI
HG03824.mapped.ILLUMINA.bwa.BEB.exome.20130415.bam	HG03824	BEB	BGI
HG03826.mapped.ILLUMINA.bwa.BEB.exome.20130415.bam	HG03826	BEB	BGI
HG03829.mapped.ILLUMINA.bwa.BEB.exome.20130415.bam	HG03829	BEB	BGI
HG03830.mapped.ILLUMINA.bwa.BEB.exome.20130415.bam	HG03830	BEB	BGI
HG03832.mapped.ILLUMINA.bwa.BEB.exome.20130415.bam	HG03832	BEB	BGI
HG03833.mapped.ILLUMINA.bwa.BEB.exome.20130415.bam	HG03833	BEB	BGI
HG03836.mapped.ILLUMINA.bwa.STU.exome.20130415.bam	HG03836	STU	BGI
HG03837.mapped.ILLUMINA.bwa.STU.exome.20130415.bam	HG03837	STU	BGI
HG03838.mapped.ILLUMINA.bwa.STU.exome.20130415.bam	HG03838	STU	BI
HG03844.mapped.ILLUMINA.bwa.STU.exome.20130415.bam	HG03844	STU	BI
HG03846.mapped.ILLUMINA.bwa.STU.exome.20130415.bam	HG03846	STU	BI
HG03848.mapped.ILLUMINA.bwa.STU.exome.20130415.bam	HG03848	STU	BI
HG03849.mapped.ILLUMINA.bwa.STU.exome.20130415.bam	HG03849	STU	BI
HG03850.mapped.ILLUMINA.bwa.STU.exome.20130415.bam	HG03850	STU	WUGSC
HG03851.mapped.ILLUMINA.bwa.STU.exome.20130415.bam	HG03851	STU	BI
HG03854.mapped.ILLUMINA.bwa.STU.exome.20130415.bam	HG03854	STU	BGI
HG03856.mapped.ILLUMINA.bwa.STU.exome.20130415.bam	HG03856	STU	BI
HG03857.mapped.ILLUMINA.bwa.STU.exome.20130415.bam	HG03857	STU	BGI
HG03858.mapped.ILLUMINA.bwa.STU.exome.20130415.bam	HG03858	STU	BI
HG03861.mapped.ILLUMINA.bwa.ITU.exome.20130415.bam	HG03861	ITU	BGI
HG03862.mapped.ILLUMINA.bwa.ITU.exome.20130415.bam	HG03862	ITU	BI
HG03863.mapped.ILLUMINA.bwa.ITU.exome.20130415.bam	HG03863	ITU	BGI
HG03864.mapped.ILLUMINA.bwa.ITU.exome.20130415.bam	HG03864	ITU	BI
HG03866.mapped.ILLUMINA.bwa.ITU.exome.20130415.bam	HG03866	ITU	WUGSC
HG03867.mapped.ILLUMINA.bwa.ITU.exome.20130415.bam	HG03867	ITU	BI
HG03868.mapped.ILLUMINA.bwa.ITU.exome.20130415.bam	HG03868	ITU	WUGSC

HG03869.mapped.ILLUMINA.bwa.ITU.exome.20130415.bam	HG03869	ITU	BI
HG03870.mapped.ILLUMINA.bwa.ITU.exome.20130415.bam	HG03870	ITU	BI
HG03871.mapped.ILLUMINA.bwa.ITU.exome.20130415.bam	HG03871	ITU	WUGSC
HG03872.mapped.ILLUMINA.bwa.ITU.exome.20130415.bam	HG03872	ITU	BI
HG03873.mapped.ILLUMINA.bwa.ITU.exome.20130415.bam	HG03873	ITU	BGI
HG03874.mapped.ILLUMINA.bwa.ITU.exome.20130415.bam	HG03874	ITU	BI
HG03875.mapped.ILLUMINA.bwa.ITU.exome.20130415.bam	HG03875	ITU	BGI
HG03882.mapped.ILLUMINA.bwa.ITU.exome.20130415.bam	HG03882	ITU	BI
HG03884.mapped.ILLUMINA.bwa.STU.exome.20130415.bam	HG03884	STU	BI
HG03885.mapped.ILLUMINA.bwa.STU.exome.20130415.bam	HG03885	STU	BI
HG03886.mapped.ILLUMINA.bwa.STU.exome.20130415.bam	HG03886	STU	BI
HG03887.mapped.ILLUMINA.bwa.STU.exome.20130415.bam	HG03887	STU	BI
HG03888.mapped.ILLUMINA.bwa.STU.exome.20130415.bam	HG03888	STU	BI
HG03890.mapped.ILLUMINA.bwa.STU.exome.20130415.bam	HG03890	STU	BI
HG03894.mapped.ILLUMINA.bwa.STU.exome.20130415.bam	HG03894	STU	WUGSC
HG03895.mapped.ILLUMINA.bwa.STU.exome.20130415.bam	HG03895	STU	BI
HG03897.mapped.ILLUMINA.bwa.STU.exome.20130415.bam	HG03897	STU	BI
HG03898.mapped.ILLUMINA.bwa.STU.exome.20130415.bam	HG03898	STU	BI
HG03899.mapped.ILLUMINA.bwa.STU.exome.20130415.bam	HG03899	STU	BGI
HG03900.mapped.ILLUMINA.bwa.STU.exome.20130415.bam	HG03900	STU	BI
HG03907.mapped.ILLUMINA.bwa.BEB.exome.20130415.bam	HG03907	BEB	BGI
HG03908.mapped.ILLUMINA.bwa.BEB.exome.20130415.bam	HG03908	BEB	BGI
HG03913.mapped.ILLUMINA.bwa.BEB.exome.20130415.bam	HG03913	BEB	BGI
HG03914.mapped.ILLUMINA.bwa.BEB.exome.20130415.bam	HG03914	BEB	BGI
HG03917.mapped.ILLUMINA.bwa.BEB.exome.20130415.bam	HG03917	BEB	BGI
HG03920.mapped.ILLUMINA.bwa.BEB.exome.20130415.bam	HG03920	BEB	BGI
HG03922.mapped.ILLUMINA.bwa.BEB.exome.20130415.bam	HG03922	BEB	BGI
HG03925.mapped.ILLUMINA.bwa.BEB.exome.20130415.bam	HG03925	BEB	BGI
HG03926.mapped.ILLUMINA.bwa.BEB.exome.20130415.bam	HG03926	BEB	BGI
HG03940.mapped.ILLUMINA.bwa.BEB.exome.20130415.bam	HG03940	BEB	BGI

HG03941.mapped.ILLUMINA.bwa.BEB.exome.20130415.bam	HG03941	BEB	BGI
HG03943.mapped.ILLUMINA.bwa.STU.exome.20130415.bam	HG03943	STU	BI
HG03944.mapped.ILLUMINA.bwa.STU.exome.20130415.bam	HG03944	STU	WUGSC
HG03945.mapped.ILLUMINA.bwa.STU.exome.20130415.bam	HG03945	STU	BGI
HG03947.mapped.ILLUMINA.bwa.STU.exome.20130415.bam	HG03947	STU	BI
HG03949.mapped.ILLUMINA.bwa.STU.exome.20130415.bam	HG03949	STU	BI
HG03950.mapped.ILLUMINA.bwa.STU.exome.20130415.bam	HG03950	STU	BI
HG03951.mapped.ILLUMINA.bwa.STU.exome.20130415.bam	HG03951	STU	BGI
HG03953.mapped.ILLUMINA.bwa.STU.exome.20130415.bam	HG03953	STU	BI
HG03955.mapped.ILLUMINA.bwa.STU.exome.20130415.bam	HG03955	STU	BGI
HG03960.mapped.ILLUMINA.bwa.ITU.exome.20130415.bam	HG03960	ITU	BI
HG03963.mapped.ILLUMINA.bwa.ITU.exome.20130415.bam	HG03963	ITU	BI
HG03965.mapped.ILLUMINA.bwa.ITU.exome.20130415.bam	HG03965	ITU	BGI
HG03967.mapped.ILLUMINA.bwa.ITU.exome.20130415.bam	HG03967	ITU	BGI
HG03968.mapped.ILLUMINA.bwa.ITU.exome.20130415.bam	HG03968	ITU	BI
HG03969.mapped.ILLUMINA.bwa.ITU.exome.20130415.bam	HG03969	ITU	WUGSC
HG03971.mapped.ILLUMINA.bwa.ITU.exome.20130415.bam	HG03971	ITU	BGI
HG03973.mapped.ILLUMINA.bwa.ITU.exome.20130415.bam	HG03973	ITU	BGI
HG03974.mapped.ILLUMINA.bwa.ITU.exome.20130415.bam	HG03974	ITU	BGI
HG03976.mapped.ILLUMINA.bwa.ITU.exome.20130415.bam	HG03976	ITU	BGI
HG03977.mapped.ILLUMINA.bwa.ITU.exome.20130415.bam	HG03977	ITU	WUGSC
HG03978.mapped.ILLUMINA.bwa.ITU.exome.20130415.bam	HG03978	ITU	BI
HG03985.mapped.ILLUMINA.bwa.STU.exome.20130415.bam	HG03985	STU	WUGSC
HG03986.mapped.ILLUMINA.bwa.STU.exome.20130415.bam	HG03986	STU	BI
HG03989.mapped.ILLUMINA.bwa.STU.exome.20130415.bam	HG03989	STU	WUGSC
HG03990.mapped.ILLUMINA.bwa.STU.exome.20130415.bam	HG03990	STU	WUGSC
HG03991.mapped.ILLUMINA.bwa.STU.exome.20130415.bam	HG03991	STU	WUGSC
HG03995.mapped.ILLUMINA.bwa.STU.exome.20130415.bam	HG03995	STU	BGI
HG03998.mapped.ILLUMINA.bwa.STU.exome.20130415.bam	HG03998	STU	WUGSC
HG03999.mapped.ILLUMINA.bwa.STU.exome.20130415.bam	HG03999	STU	BGI

HG04001.mapped.ILLUMINA.bwa.ITU.exome.20130415.bam	HG04001	ITU	WUGSC
HG04002.mapped.ILLUMINA.bwa.ITU.exome.20130415.bam	HG04002	ITU	BGI
HG04003.mapped.ILLUMINA.bwa.STU.exome.20130415.bam	HG04003	STU	WUGSC
HG04014.mapped.ILLUMINA.bwa.ITU.exome.20130415.bam	HG04014	ITU	BI
HG04015.mapped.ILLUMINA.bwa.ITU.exome.20130415.bam	HG04015	ITU	BI
HG04017.mapped.ILLUMINA.bwa.ITU.exome.20130415.bam	HG04017	ITU	BGI
HG04018.mapped.ILLUMINA.bwa.ITU.exome.20130415.bam	HG04018	ITU	BGI
HG04019.mapped.ILLUMINA.bwa.ITU.exome.20130415.bam	HG04019	ITU	BI
HG04020.mapped.ILLUMINA.bwa.ITU.exome.20130415.bam	HG04020	ITU	BI
HG04022.mapped.ILLUMINA.bwa.ITU.exome.20130415.bam	HG04022	ITU	BI
HG04023.mapped.ILLUMINA.bwa.ITU.exome.20130415.bam	HG04023	ITU	WUGSC
HG04025.mapped.ILLUMINA.bwa.ITU.exome.20130415.bam	HG04025	ITU	BI
HG04026.mapped.ILLUMINA.bwa.ITU.exome.20130415.bam	HG04026	ITU	BGI
HG04029.mapped.ILLUMINA.bwa.STU.exome.20130415.bam	HG04029	STU	BI
HG04033.mapped.ILLUMINA.bwa.STU.exome.20130415.bam	HG04033	STU	BI
HG04035.mapped.ILLUMINA.bwa.STU.exome.20130415.bam	HG04035	STU	BI
HG04038.mapped.ILLUMINA.bwa.STU.exome.20130415.bam	HG04038	STU	WUGSC
HG04039.mapped.ILLUMINA.bwa.STU.exome.20130415.bam	HG04039	STU	WUGSC
HG04042.mapped.ILLUMINA.bwa.STU.exome.20130415.bam	HG04042	STU	BI
HG04047.mapped.ILLUMINA.bwa.STU.exome.20130415.bam	HG04047	STU	WUGSC
HG04054.mapped.ILLUMINA.bwa.ITU.exome.20130415.bam	HG04054	ITU	WUGSC
HG04056.mapped.ILLUMINA.bwa.ITU.exome.20130415.bam	HG04056	ITU	WUGSC
HG04059.mapped.ILLUMINA.bwa.ITU.exome.20130415.bam	HG04059	ITU	WUGSC
HG04060.mapped.ILLUMINA.bwa.ITU.exome.20130415.bam	HG04060	ITU	WUGSC
HG04061.mapped.ILLUMINA.bwa.ITU.exome.20130415.bam	HG04061	ITU	WUGSC
HG04062.mapped.ILLUMINA.bwa.ITU.exome.20130415.bam	HG04062	ITU	WUGSC
HG04063.mapped.ILLUMINA.bwa.ITU.exome.20130415.bam	HG04063	ITU	BI
HG04070.mapped.ILLUMINA.bwa.ITU.exome.20130415.bam	HG04070	ITU	BGI
HG04075.mapped.ILLUMINA.bwa.STU.exome.20130415.bam	HG04075	STU	BGI
HG04076.mapped.ILLUMINA.bwa.ITU.exome.20130415.bam	HG04076	ITU	WUGSC

HG04080.mapped.ILLUMINA.bwa.ITU.exome.20130415.bam	HG04080	ITU	WUGSC
HG04090.mapped.ILLUMINA.bwa.ITU.exome.20130415.bam	HG04090	ITU	WUGSC
HG04093.mapped.ILLUMINA.bwa.ITU.exome.20130415.bam	HG04093	ITU	BI
HG04094.mapped.ILLUMINA.bwa.ITU.exome.20130415.bam	HG04094	ITU	WUGSC
HG04096.mapped.ILLUMINA.bwa.ITU.exome.20130415.bam	HG04096	ITU	BGI
HG04098.mapped.ILLUMINA.bwa.ITU.exome.20130415.bam	HG04098	ITU	BGI
HG04099.mapped.ILLUMINA.bwa.STU.exome.20130415.bam	HG04099	STU	BI
HG04100.mapped.ILLUMINA.bwa.STU.exome.20130415.bam	HG04100	STU	BGI
HG04106.mapped.ILLUMINA.bwa.STU.exome.20130415.bam	HG04106	STU	BI
HG04107.mapped.ILLUMINA.bwa.STU.exome.20130415.bam	HG04107	STU	BI
HG04118.mapped.ILLUMINA.bwa.ITU.exome.20130415.bam	HG04118	ITU	WUGSC
HG04131.mapped.ILLUMINA.bwa.BEB.exome.20130415.bam	HG04131	BEB	WUGSC
HG04134.mapped.ILLUMINA.bwa.BEB.exome.20130415.bam	HG04134	BEB	WUGSC
HG04140.mapped.ILLUMINA.bwa.BEB.exome.20130415.bam	HG04140	BEB	WUGSC
HG04141.mapped.ILLUMINA.bwa.BEB.exome.20130415.bam	HG04141	BEB	WUGSC
HG04144.mapped.ILLUMINA.bwa.BEB.exome.20130415.bam	HG04144	BEB	BGI
HG04152.mapped.ILLUMINA.bwa.BEB.exome.20130415.bam	HG04152	BEB	BGI
HG04153.mapped.ILLUMINA.bwa.BEB.exome.20130415.bam	HG04153	BEB	BGI
HG04155.mapped.ILLUMINA.bwa.BEB.exome.20130415.bam	HG04155	BEB	BGI
HG04156.mapped.ILLUMINA.bwa.BEB.exome.20130415.bam	HG04156	BEB	BGI
HG04158.mapped.ILLUMINA.bwa.BEB.exome.20130415.bam	HG04158	BEB	WUGSC
HG04161.mapped.ILLUMINA.bwa.BEB.exome.20130415.bam	HG04161	BEB	BGI
HG04162.mapped.ILLUMINA.bwa.BEB.exome.20130415.bam	HG04162	BEB	BGI
HG04164.mapped.ILLUMINA.bwa.BEB.exome.20130415.bam	HG04164	BEB	BGI
HG04171.mapped.ILLUMINA.bwa.BEB.exome.20130415.bam	HG04171	BEB	BGI
HG04173.mapped.ILLUMINA.bwa.BEB.exome.20130415.bam	HG04173	BEB	WUGSC
HG04176.mapped.ILLUMINA.bwa.BEB.exome.20130415.bam	HG04176	BEB	BGI
HG04177.mapped.ILLUMINA.bwa.BEB.exome.20130415.bam	HG04177	BEB	BGI
HG04180.mapped.ILLUMINA.bwa.BEB.exome.20130415.bam	HG04180	BEB	BGI
HG04182.mapped.ILLUMINA.bwa.BEB.exome.20130415.bam	HG04182	BEB	WUGSC

HG04183.mapped.ILLUMINA.bwa.BEB.exome.20130415.bam	HG04183	BEB	WUGSC
HG04185.mapped.ILLUMINA.bwa.BEB.exome.20130415.bam	HG04185	BEB	WUGSC
HG04186.mapped.ILLUMINA.bwa.BEB.exome.20130415.bam	HG04186	BEB	WUGSC
HG04188.mapped.ILLUMINA.bwa.BEB.exome.20130415.bam	HG04188	BEB	BGI
HG04189.mapped.ILLUMINA.bwa.BEB.exome.20130415.bam	HG04189	BEB	BGI
HG04194.mapped.ILLUMINA.bwa.BEB.exome.20130415.bam	HG04194	BEB	BGI
HG04195.mapped.ILLUMINA.bwa.BEB.exome.20130415.bam	HG04195	BEB	BGI
HG04198.mapped.ILLUMINA.bwa.ITU.exome.20130415.bam	HG04198	ITU	BGI
HG04200.mapped.ILLUMINA.bwa.ITU.exome.20130415.bam	HG04200	ITU	BGI
HG04202.mapped.ILLUMINA.bwa.ITU.exome.20130415.bam	HG04202	ITU	BGI
HG04206.mapped.ILLUMINA.bwa.ITU.exome.20130415.bam	HG04206	ITU	BGI
HG04209.mapped.ILLUMINA.bwa.ITU.exome.20130415.bam	HG04209	ITU	BGI
HG04210.mapped.ILLUMINA.bwa.STU.exome.20130415.bam	HG04210	STU	BGI
HG04211.mapped.ILLUMINA.bwa.ITU.exome.20130415.bam	HG04211	ITU	BGI
HG04212.mapped.ILLUMINA.bwa.ITU.exome.20130415.bam	HG04212	ITU	BGI
HG04214.mapped.ILLUMINA.bwa.ITU.exome.20130415.bam	HG04214	ITU	BGI
HG04216.mapped.ILLUMINA.bwa.ITU.exome.20130415.bam	HG04216	ITU	BGI
HG04219.mapped.ILLUMINA.bwa.ITU.exome.20130415.bam	HG04219	ITU	BGI
HG04222.mapped.ILLUMINA.bwa.ITU.exome.20130415.bam	HG04222	ITU	BGI
HG04225.mapped.ILLUMINA.bwa.ITU.exome.20130415.bam	HG04225	ITU	BGI
HG04227.mapped.ILLUMINA.bwa.STU.exome.20130415.bam	HG04227	STU	BGI
HG04229.mapped.ILLUMINA.bwa.STU.exome.20130415.bam	HG04229	STU	BGI
HG04235.mapped.ILLUMINA.bwa.ITU.exome.20130415.bam	HG04235	ITU	BGI
HG04238.mapped.ILLUMINA.bwa.ITU.exome.20130415.bam	HG04238	ITU	BGI
HG04239.mapped.ILLUMINA.bwa.ITU.exome.20130415.bam	HG04239	ITU	BGI
NA06985.mapped.ILLUMINA.bwa.CEU.exome.20130415.bam	NA06985	CEU	BCM
NA07000.mapped.ILLUMINA.bwa.CEU.exome.20130415.bam	NA07000	CEU	BCM
NA07056.mapped.ILLUMINA.bwa.CEU.exome.20130415.bam	NA07056	CEU	BCM
NA07357.mapped.ILLUMINA.bwa.CEU.exome.20130415.bam	NA07357	CEU	BCM
NA10851.mapped.ILLUMINA.bwa.CEU.exome.20130415.bam	NA10851	CEU	BCM

NA11829.mapped.ILLUMINA.bwa.CEU.exome.20130415.bam	NA11829	CEU	BCM
NA11830.mapped.ILLUMINA.bwa.CEU.exome.20130415.bam	NA11830	CEU	BCM
NA11831.mapped.ILLUMINA.bwa.CEU.exome.20130415.bam	NA11831	CEU	BCM
NA11832.mapped.ILLUMINA.bwa.CEU.exome.20130415.bam	NA11832	CEU	BCM
NA11881.mapped.ILLUMINA.bwa.CEU.exome.20130415.bam	NA11881	CEU	BCM
NA11992.mapped.ILLUMINA.bwa.CEU.exome.20130415.bam	NA11992	CEU	BCM
NA11994.mapped.ILLUMINA.bwa.CEU.exome.20130415.bam	NA11994	CEU	BCM
NA11995.mapped.ILLUMINA.bwa.CEU.exome.20130415.bam	NA11995	CEU	BCM
NA12003.mapped.ILLUMINA.bwa.CEU.exome.20130415.bam	NA12003	CEU	BCM
NA12004.mapped.ILLUMINA.bwa.CEU.exome.20130415.bam	NA12004	CEU	BCM
NA12005.mapped.ILLUMINA.bwa.CEU.exome.20130415.bam	NA12005	CEU	BCM
NA12006.mapped.ILLUMINA.bwa.CEU.exome.20130415.bam	NA12006	CEU	BCM
NA12043.mapped.ILLUMINA.bwa.CEU.exome.20130415.bam	NA12043	CEU	BCM
NA12044.mapped.ILLUMINA.bwa.CEU.exome.20130415.bam	NA12044	CEU	BCM
NA12046.mapped.ILLUMINA.bwa.CEU.exome.20130415.bam	NA12046	CEU	BCM
NA12144.mapped.ILLUMINA.bwa.CEU.exome.20130415.bam	NA12144	CEU	BCM
NA12154.mapped.ILLUMINA.bwa.CEU.exome.20130415.bam	NA12154	CEU	BCM
NA12155.mapped.ILLUMINA.bwa.CEU.exome.20130415.bam	NA12155	CEU	BCM
NA12156.mapped.ILLUMINA.bwa.CEU.exome.20130415.bam	NA12156	CEU	BCM
NA12234.mapped.ILLUMINA.bwa.CEU.exome.20130415.bam	NA12234	CEU	BCM
NA12414.mapped.ILLUMINA.bwa.CEU.exome.20130415.bam	NA12414	CEU	BCM
NA12489.mapped.ILLUMINA.bwa.CEU.exome.20130415.bam	NA12489	CEU	BCM
NA12750.mapped.ILLUMINA.bwa.CEU.exome.20130415.bam	NA12750	CEU	WUGSC
NA12762.mapped.ILLUMINA.bwa.CEU.exome.20130415.bam	NA12762	CEU	BCM
NA12812.mapped.ILLUMINA.bwa.CEU.exome.20130415.bam	NA12812	CEU	BCM
NA12813.mapped.ILLUMINA.bwa.CEU.exome.20130415.bam	NA12813	CEU	BCM
NA12814.mapped.ILLUMINA.bwa.CEU.exome.20130415.bam	NA12814	CEU	BCM
NA12815.mapped.ILLUMINA.bwa.CEU.exome.20130415.bam	NA12815	CEU	BCM
NA12872.mapped.ILLUMINA.bwa.CEU.exome.20130415.bam	NA12872	CEU	BCM
NA12873.mapped.ILLUMINA.bwa.CEU.exome.20130415.bam	NA12873	CEU	BCM

NA12874.mapped.ILLUMINA.bwa.CEU.exome.20130415.bam	NA12874	CEU	BCM
NA18502.mapped.ILLUMINA.bwa.YRI.exome.20130415.bam	NA18502	YRI	BCM
NA18505.mapped.ILLUMINA.bwa.YRI.exome.20130415.bam	NA18505	YRI	BCM
NA18507.mapped.ILLUMINA.bwa.YRI.exome.20130415.bam	NA18507	YRI	BCM
NA18508.mapped.ILLUMINA.bwa.YRI.exome.20130415.bam	NA18508	YRI	BCM
NA18525.mapped.ILLUMINA.bwa.CHB.exome.20130415.bam	NA18525	CHB	BI
NA18528.mapped.ILLUMINA.bwa.CHB.exome.20130415.bam	NA18528	CHB	BCM
NA18531.mapped.ILLUMINA.bwa.CHB.exome.20130415.bam	NA18531	CHB	BCM
NA18591.mapped.ILLUMINA.bwa.CHB.exome.20130415.bam	NA18591	CHB	WUGSC
NA18614.mapped.ILLUMINA.bwa.CHB.exome.20130415.bam	NA18614	CHB	BCM
NA18615.mapped.ILLUMINA.bwa.CHB.exome.20130415.bam	NA18615	CHB	BCM
NA18617.mapped.ILLUMINA.bwa.CHB.exome.20130415.bam	NA18617	CHB	BCM
NA18618.mapped.ILLUMINA.bwa.CHB.exome.20130415.bam	NA18618	CHB	BCM
NA18619.mapped.ILLUMINA.bwa.CHB.exome.20130415.bam	NA18619	CHB	BCM
NA18625.mapped.ILLUMINA.bwa.CHB.exome.20130415.bam	NA18625	CHB	BCM
NA18626.mapped.ILLUMINA.bwa.CHB.exome.20130415.bam	NA18626	CHB	BCM
NA18627.mapped.ILLUMINA.bwa.CHB.exome.20130415.bam	NA18627	CHB	BCM
NA18628.mapped.ILLUMINA.bwa.CHB.exome.20130415.bam	NA18628	CHB	BCM
NA18629.mapped.ILLUMINA.bwa.CHB.exome.20130415.bam	NA18629	CHB	WUGSC
NA18630.mapped.ILLUMINA.bwa.CHB.exome.20130415.bam	NA18630	CHB	BCM
NA18639.mapped.ILLUMINA.bwa.CHB.exome.20130415.bam	NA18639	CHB	BCM
NA18640.mapped.ILLUMINA.bwa.CHB.exome.20130415.bam	NA18640	CHB	BCM
NA18641.mapped.ILLUMINA.bwa.CHB.exome.20130415.bam	NA18641	CHB	BCM
NA18642.mapped.ILLUMINA.bwa.CHB.exome.20130415.bam	NA18642	CHB	BCM
NA18643.mapped.ILLUMINA.bwa.CHB.exome.20130415.bam	NA18643	CHB	BCM
NA18644.mapped.ILLUMINA.bwa.CHB.exome.20130415.bam	NA18644	CHB	WUGSC
NA18645.mapped.ILLUMINA.bwa.CHB.exome.20130415.bam	NA18645	CHB	BCM
NA18646.mapped.ILLUMINA.bwa.CHB.exome.20130415.bam	NA18646	CHB	WUGSC
NA18647.mapped.ILLUMINA.bwa.CHB.exome.20130415.bam	NA18647	CHB	BCM
NA18648.mapped.ILLUMINA.bwa.CHB.exome.20130415.bam	NA18648	CHB	WUGSC

NA18740.mapped.ILLUMINA.bwa.CHB.exome.20130415.bam	NA18740	CHB	BCM
NA18745.mapped.ILLUMINA.bwa.CHB.exome.20130415.bam	NA18745	CHB	BCM
NA18747.mapped.ILLUMINA.bwa.CHB.exome.20130415.bam	NA18747	CHB	BCM
NA18748.mapped.ILLUMINA.bwa.CHB.exome.20130415.bam	NA18748	CHB	BCM
NA18749.mapped.ILLUMINA.bwa.CHB.exome.20130415.bam	NA18749	CHB	BCM
NA18757.mapped.ILLUMINA.bwa.CHB.exome.20130415.bam	NA18757	CHB	BCM
NA18864.mapped.ILLUMINA.bwa.YRI.exome.20130415.bam	NA18864	YRI	BGI
NA18865.mapped.ILLUMINA.bwa.YRI.exome.20130415.bam	NA18865	YRI	BGI
NA18876.mapped.ILLUMINA.bwa.YRI.exome.20130415.bam	NA18876	YRI	BGI
NA18877.mapped.ILLUMINA.bwa.YRI.exome.20130415.bam	NA18877	YRI	BGI
NA18878.mapped.ILLUMINA.bwa.YRI.exome.20130415.bam	NA18878	YRI	BGI
NA18879.mapped.ILLUMINA.bwa.YRI.exome.20130415.bam	NA18879	YRI	BGI
NA18881.mapped.ILLUMINA.bwa.YRI.exome.20130415.bam	NA18881	YRI	BGI
NA18915.mapped.ILLUMINA.bwa.YRI.exome.20130415.bam	NA18915	YRI	BGI
NA18939.mapped.ILLUMINA.bwa.JPT.exome.20130415.bam	NA18939	JPT	BI
NA18941.mapped.ILLUMINA.bwa.JPT.exome.20130415.bam	NA18941	JPT	BCM
NA18946.mapped.ILLUMINA.bwa.JPT.exome.20130415.bam	NA18946	JPT	BCM
NA18954.mapped.ILLUMINA.bwa.JPT.exome.20130415.bam	NA18954	JPT	BCM
NA18956.mapped.ILLUMINA.bwa.JPT.exome.20130415.bam	NA18956	JPT	BCM
NA18957.mapped.ILLUMINA.bwa.JPT.exome.20130415.bam	NA18957	JPT	BCM
NA18962.mapped.ILLUMINA.bwa.JPT.exome.20130415.bam	NA18962	JPT	BCM
NA18963.mapped.ILLUMINA.bwa.JPT.exome.20130415.bam	NA18963	JPT	BCM
NA18965.mapped.ILLUMINA.bwa.JPT.exome.20130415.bam	NA18965	JPT	BCM
NA18977.mapped.ILLUMINA.bwa.JPT.exome.20130415.bam	NA18977	JPT	BCM
NA18978.mapped.ILLUMINA.bwa.JPT.exome.20130415.bam	NA18978	JPT	BCM
NA18979.mapped.ILLUMINA.bwa.JPT.exome.20130415.bam	NA18979	JPT	BCM
NA18980.mapped.ILLUMINA.bwa.JPT.exome.20130415.bam	NA18980	JPT	BCM
NA18992.mapped.ILLUMINA.bwa.JPT.exome.20130415.bam	NA18992	JPT	BCM
NA18993.mapped.ILLUMINA.bwa.JPT.exome.20130415.bam	NA18993	JPT	BCM
NA18994.mapped.ILLUMINA.bwa.JPT.exome.20130415.bam	NA18994	JPT	BCM

NA18995.mapped.ILLUMINA.bwa.JPT.exome.20130415.bam	NA18995	JPT	BCM
NA18997.mapped.ILLUMINA.bwa.JPT.exome.20130415.bam	NA18997	JPT	BCM
NA18998.mapped.ILLUMINA.bwa.JPT.exome.20130415.bam	NA18998	JPT	BCM
NA19001.mapped.ILLUMINA.bwa.JPT.exome.20130415.bam	NA19001	JPT	BCM
NA19002.mapped.ILLUMINA.bwa.JPT.exome.20130415.bam	NA19002	JPT	BCM
NA19005.mapped.ILLUMINA.bwa.JPT.exome.20130415.bam	NA19005	JPT	BCM
NA19006.mapped.ILLUMINA.bwa.JPT.exome.20130415.bam	NA19006	JPT	BGI
NA19009.mapped.ILLUMINA.bwa.JPT.exome.20130415.bam	NA19009	JPT	BCM
NA19010.mapped.ILLUMINA.bwa.JPT.exome.20130415.bam	NA19010	JPT	BCM
NA19011.mapped.ILLUMINA.bwa.JPT.exome.20130415.bam	NA19011	JPT	BGI
NA19025.mapped.ILLUMINA.bwa.LWK.exome.20130415.bam	NA19025	LWK	BGI
NA19027.mapped.ILLUMINA.bwa.LWK.exome.20130415.bam	NA19027	LWK	BCM
NA19037.mapped.ILLUMINA.bwa.LWK.exome.20130415.bam	NA19037	LWK	BGI
NA19038.mapped.ILLUMINA.bwa.LWK.exome.20130415.bam	NA19038	LWK	BCM
NA19041.mapped.ILLUMINA.bwa.LWK.exome.20130415.bam	NA19041	LWK	BCM
NA19042.mapped.ILLUMINA.bwa.LWK.exome.20130415.bam	NA19042	LWK	BGI
NA19078.mapped.ILLUMINA.bwa.JPT.exome.20130415.bam	NA19078	JPT	WUGSC
NA19089.mapped.ILLUMINA.bwa.JPT.exome.20130415.bam	NA19089	JPT	BGI
NA19090.mapped.ILLUMINA.bwa.JPT.exome.20130415.bam	NA19090	JPT	BGI
NA19091.mapped.ILLUMINA.bwa.JPT.exome.20130415.bam	NA19091	JPT	BGI
NA19095.mapped.ILLUMINA.bwa.YRI.exome.20130415.bam	NA19095	YRI	BCM
NA19113.mapped.ILLUMINA.bwa.YRI.exome.20130415.bam	NA19113	YRI	BCM
NA19117.mapped.ILLUMINA.bwa.YRI.exome.20130415.bam	NA19117	YRI	BCM
NA19118.mapped.ILLUMINA.bwa.YRI.exome.20130415.bam	NA19118	YRI	BCM
NA19121.mapped.ILLUMINA.bwa.YRI.exome.20130415.bam	NA19121	YRI	BCM
NA19137.mapped.ILLUMINA.bwa.YRI.exome.20130415.bam	NA19137	YRI	WUGSC
NA19149.mapped.ILLUMINA.bwa.YRI.exome.20130415.bam	NA19149	YRI	BCM
NA19184.mapped.ILLUMINA.bwa.YRI.exome.20130415.bam	NA19184	YRI	BCM
NA19185.mapped.ILLUMINA.bwa.YRI.exome.20130415.bam	NA19185	YRI	BCM
NA19238.mapped.ILLUMINA.bwa.YRI.exome.20130415.bam	NA19238	YRI	WUGSC

NA19239.mapped.ILLUMINA.bwa.YRI.exome.20130415.bam	NA19239	YRI	WUGSC
NA19240.mapped.ILLUMINA.bwa.YRI.exome.20130415.bam	NA19240	YRI	BI
NA19307.mapped.ILLUMINA.bwa.LWK.exome.20130415.bam	NA19307	LWK	BCM
NA19309.mapped.ILLUMINA.bwa.LWK.exome.20130415.bam	NA19309	LWK	BCM
NA19320.mapped.ILLUMINA.bwa.LWK.exome.20130415.bam	NA19320	LWK	BGI
NA19323.mapped.ILLUMINA.bwa.LWK.exome.20130415.bam	NA19323	LWK	BI
NA19351.mapped.ILLUMINA.bwa.LWK.exome.20130415.bam	NA19351	LWK	BCM
NA19355.mapped.ILLUMINA.bwa.LWK.exome.20130415.bam	NA19355	LWK	BCM
NA19360.mapped.ILLUMINA.bwa.LWK.exome.20130415.bam	NA19360	LWK	BCM
NA19374.mapped.ILLUMINA.bwa.LWK.exome.20130415.bam	NA19374	LWK	BCM
NA19376.mapped.ILLUMINA.bwa.LWK.exome.20130415.bam	NA19376	LWK	BCM
NA19378.mapped.ILLUMINA.bwa.LWK.exome.20130415.bam	NA19378	LWK	BI
NA19379.mapped.ILLUMINA.bwa.LWK.exome.20130415.bam	NA19379	LWK	BCM
NA19390.mapped.ILLUMINA.bwa.LWK.exome.20130415.bam	NA19390	LWK	BCM
NA19391.mapped.ILLUMINA.bwa.LWK.exome.20130415.bam	NA19391	LWK	BCM
NA19401.mapped.ILLUMINA.bwa.LWK.exome.20130415.bam	NA19401	LWK	BCM
NA19403.mapped.ILLUMINA.bwa.LWK.exome.20130415.bam	NA19403	LWK	BCM
NA19430.mapped.ILLUMINA.bwa.LWK.exome.20130415.bam	NA19430	LWK	BCM
NA19435.mapped.ILLUMINA.bwa.LWK.exome.20130415.bam	NA19435	LWK	BCM
NA19436.mapped.ILLUMINA.bwa.LWK.exome.20130415.bam	NA19436	LWK	BCM
NA19437.mapped.ILLUMINA.bwa.LWK.exome.20130415.bam	NA19437	LWK	BCM
NA19438.mapped.ILLUMINA.bwa.LWK.exome.20130415.bam	NA19438	LWK	BCM
NA19445.mapped.ILLUMINA.bwa.LWK.exome.20130415.bam	NA19445	LWK	BCM
NA19446.mapped.ILLUMINA.bwa.LWK.exome.20130415.bam	NA19446	LWK	BCM
NA19448.mapped.ILLUMINA.bwa.LWK.exome.20130415.bam	NA19448	LWK	BCM
NA19454.mapped.ILLUMINA.bwa.LWK.exome.20130415.bam	NA19454	LWK	BI
NA19467.mapped.ILLUMINA.bwa.LWK.exome.20130415.bam	NA19467	LWK	BCM
NA19475.mapped.ILLUMINA.bwa.LWK.exome.20130415.bam	NA19475	LWK	BGI
NA19740.mapped.ILLUMINA.bwa.MXL.exome.20130415.bam	NA19740	MXL	BCM
NA19749.mapped.ILLUMINA.bwa.MXL.exome.20130415.bam	NA19749	MXL	WUGSC

NA19764.mapped.ILLUMINA.bwa.MXL.exome.20130415.bam	NA19764	MXL	BCM
NA19795.mapped.ILLUMINA.bwa.MXL.exome.20130415.bam	NA19795	MXL	BCM
NA19913.mapped.ILLUMINA.bwa.ASW.exome.20130415.bam	NA19913	ASW	BGI
NA19922.mapped.ILLUMINA.bwa.ASW.exome.20130415.bam	NA19922	ASW	BCM
NA19923.mapped.ILLUMINA.bwa.ASW.exome.20130415.bam	NA19923	ASW	BCM
NA20274.mapped.ILLUMINA.bwa.ASW.exome.20130415.bam	NA20274	ASW	BGI
NA20298.mapped.ILLUMINA.bwa.ASW.exome.20130415.bam	NA20298	ASW	BCM
NA20318.mapped.ILLUMINA.bwa.ASW.exome.20130415.bam	NA20318	ASW	BGI
NA20320.mapped.ILLUMINA.bwa.ASW.exome.20130415.bam	NA20320	ASW	BGI
NA20321.mapped.ILLUMINA.bwa.ASW.exome.20130415.bam	NA20321	ASW	BGI
NA20339.mapped.ILLUMINA.bwa.ASW.exome.20130415.bam	NA20339	ASW	BCM
NA20351.mapped.ILLUMINA.bwa.ASW.exome.20130415.bam	NA20351	ASW	BCM
NA20355.mapped.ILLUMINA.bwa.ASW.exome.20130415.bam	NA20355	ASW	BGI
NA20362.mapped.ILLUMINA.bwa.ASW.exome.20130415.bam	NA20362	ASW	WUGSC
NA20412.mapped.ILLUMINA.bwa.ASW.exome.20130415.bam	NA20412	ASW	BCM
NA20502.mapped.ILLUMINA.bwa.TSI.exome.20130415.bam	NA20502	TSI	BI
NA20503.mapped.ILLUMINA.bwa.TSI.exome.20130415.bam	NA20503	TSI	BCM
NA20505.mapped.ILLUMINA.bwa.TSI.exome.20130415.bam	NA20505	TSI	BCM
NA20508.mapped.ILLUMINA.bwa.TSI.exome.20130415.bam	NA20508	TSI	BCM
NA20511.mapped.ILLUMINA.bwa.TSI.exome.20130415.bam	NA20511	TSI	BGI
NA20512.mapped.ILLUMINA.bwa.TSI.exome.20130415.bam	NA20512	TSI	BCM
NA20516.mapped.ILLUMINA.bwa.TSI.exome.20130415.bam	NA20516	TSI	BCM
NA20517.mapped.ILLUMINA.bwa.TSI.exome.20130415.bam	NA20517	TSI	BCM
NA20524.mapped.ILLUMINA.bwa.TSI.exome.20130415.bam	NA20524	TSI	BCM
NA20526.mapped.ILLUMINA.bwa.TSI.exome.20130415.bam	NA20526	TSI	BCM
NA20528.mapped.ILLUMINA.bwa.TSI.exome.20130415.bam	NA20528	TSI	BCM
NA20529.mapped.ILLUMINA.bwa.TSI.exome.20130415.bam	NA20529	TSI	BCM
NA20530.mapped.ILLUMINA.bwa.TSI.exome.20130415.bam	NA20530	TSI	BCM
NA20531.mapped.ILLUMINA.bwa.TSI.exome.20130415.bam	NA20531	TSI	BCM
NA20532.mapped.ILLUMINA.bwa.TSI.exome.20130415.bam	NA20532	TSI	BCM

NA20533.mapped.ILLUMINA.bwa.TSI.exome.20130415.bam	NA20533	TSI	BCM
NA20534.mapped.ILLUMINA.bwa.TSI.exome.20130415.bam	NA20534	TSI	BCM
NA20535.mapped.ILLUMINA.bwa.TSI.exome.20130415.bam	NA20535	TSI	BCM
NA20536.mapped.ILLUMINA.bwa.TSI.exome.20130415.bam	NA20536	TSI	BCM
NA20538.mapped.ILLUMINA.bwa.TSI.exome.20130415.bam	NA20538	TSI	BCM
NA20539.mapped.ILLUMINA.bwa.TSI.exome.20130415.bam	NA20539	TSI	BCM
NA20540.mapped.ILLUMINA.bwa.TSI.exome.20130415.bam	NA20540	TSI	BCM
NA20541.mapped.ILLUMINA.bwa.TSI.exome.20130415.bam	NA20541	TSI	BCM
NA20762.mapped.ILLUMINA.bwa.TSI.exome.20130415.bam	NA20762	TSI	BGI
NA20763.mapped.ILLUMINA.bwa.TSI.exome.20130415.bam	NA20763	TSI	BGI
NA20764.mapped.ILLUMINA.bwa.TSI.exome.20130415.bam	NA20764	TSI	BGI
NA20767.mapped.ILLUMINA.bwa.TSI.exome.20130415.bam	NA20767	TSI	BGI
NA20821.mapped.ILLUMINA.bwa.TSI.exome.20130415.bam	NA20821	TSI	BGI
NA20822.mapped.ILLUMINA.bwa.TSI.exome.20130415.bam	NA20822	TSI	BGI
NA20827.mapped.ILLUMINA.bwa.TSI.exome.20130415.bam	NA20827	TSI	WUGSC
NA20832.mapped.ILLUMINA.bwa.TSI.exome.20130415.bam	NA20832	TSI	WUGSC
NA20882.mapped.ILLUMINA.bwa.GIH.exome.20130415.bam	NA20882	GIH	BCM
NA20884.mapped.ILLUMINA.bwa.GIH.exome.20130415.bam	NA20884	GIH	BCM
NA20900.mapped.ILLUMINA.bwa.GIH.exome.20130415.bam	NA20900	GIH	BGI
NA21104.mapped.ILLUMINA.bwa.GIH.exome.20130415.bam	NA21104	GIH	BCM
NA21105.mapped.ILLUMINA.bwa.GIH.exome.20130415.bam	NA21105	GIH	BCM
NA21106.mapped.ILLUMINA.bwa.GIH.exome.20130415.bam	NA21106	GIH	BCM
NA21107.mapped.ILLUMINA.bwa.GIH.exome.20130415.bam	NA21107	GIH	BCM
NA21108.mapped.ILLUMINA.bwa.GIH.exome.20130415.bam	NA21108	GIH	BCM
NA21109.mapped.ILLUMINA.bwa.GIH.exome.20130415.bam	NA21109	GIH	BCM
NA21110.mapped.ILLUMINA.bwa.GIH.exome.20130415.bam	NA21110	GIH	BCM
NA21111.mapped.ILLUMINA.bwa.GIH.exome.20130415.bam	NA21111	GIH	BCM
NA21112.mapped.ILLUMINA.bwa.GIH.exome.20130415.bam	NA21112	GIH	BCM
NA21113.mapped.ILLUMINA.bwa.GIH.exome.20130415.bam	NA21113	GIH	BCM
NA21115.mapped.ILLUMINA.bwa.GIH.exome.20130415.bam	NA21115	GIH	BCM

NA21116.mapped.ILLUMINA.bwa.GIH.exome.20130415.bam	NA21116	GIH	BCM
NA21117.mapped.ILLUMINA.bwa.GIH.exome.20130415.bam	NA21117	GIH	BCM
NA21118.mapped.ILLUMINA.bwa.GIH.exome.20130415.bam	NA21118	GIH	BCM
NA21119.mapped.ILLUMINA.bwa.GIH.exome.20130415.bam	NA21119	GIH	BCM
NA21120.mapped.ILLUMINA.bwa.GIH.exome.20130415.bam	NA21120	GIH	BCM
NA21122.mapped.ILLUMINA.bwa.GIH.exome.20130415.bam	NA21122	GIH	BCM
NA21123.mapped.ILLUMINA.bwa.GIH.exome.20130415.bam	NA21123	GIH	BCM
NA21124.mapped.ILLUMINA.bwa.GIH.exome.20130415.bam	NA21124	GIH	BI
NA21125.mapped.ILLUMINA.bwa.GIH.exome.20130415.bam	NA21125	GIH	BCM
NA21127.mapped.ILLUMINA.bwa.GIH.exome.20130415.bam	NA21127	GIH	BGI
NA21128.mapped.ILLUMINA.bwa.GIH.exome.20130415.bam	NA21128	GIH	BGI
NA21129.mapped.ILLUMINA.bwa.GIH.exome.20130415.bam	NA21129	GIH	BI
NA21130.mapped.ILLUMINA.bwa.GIH.exome.20130415.bam	NA21130	GIH	BGI
NA21133.mapped.ILLUMINA.bwa.GIH.exome.20130415.bam	NA21133	GIH	BGI
NA21135.mapped.ILLUMINA.bwa.GIH.exome.20130415.bam	NA21135	GIH	BGI
