# Supplementary material for: Methodological differences can affect sequencing depth with a possible impact on the accuracy of genetic diagnosis
Source: Genet Mol Biol. 2020 Apr 27;43(2):e20190270. doi: 10.1590/1678-4685-GMB-2019-0270 (PMC7198014; doi:10.1590/1678-4685-GMB-2019-0270)
Supplement: File S3. [file 1415-4757-GMB-43-2-e20190270-s3.zip › gmb-2019-0270_20200217_suppl3.html]

Methodological differences can affect sequencing depth with possible impact on the accuracy of genetic diagnosis


# Methodological differences can affect sequencing depth with possible impact on the accuracy of genetic diagnosis

#### *Murilo G Borges, Cristiane S Rocha, Benilton S Carvalho and Iscia Lopes-Cendes*

# Supplementary file 3

## Depth distribution for sequencing centers

Figure S1-1: Depth distribution varies significantly ( \(\le\) 0.001) among the four sequencing centers considered in this study (BCM - Baylor College of Medicine, BI - Broad Institute, BGI and WUGC - Washington University Genome Center). They show an average of 82.8 \(\pm\) 67.6 for BCM, 123.0 \(\pm\) 85.6 for BGI, 86.6 \(\pm\) 79.2 for BI and 49.4 \(\pm\) 33.8 for WUGSC.

## PCA for sequencing centers

Summary for PCA analysis:

```
##                             PC1      PC2      PC3
## Standard deviation     24.20456 13.50666 13.01586
## Proportion of Variance  0.52685  0.16406  0.15235
## Cumulative Proportion   0.52685  0.69091  0.84326
```

Figure S1-2: Principal component analysis for the depth of the four sequencing centers considered in this study (BCM - Baylor College of Medicine, BI - Broad Institute, BGI and WUGC - Washington University Genome Center) corroborates our findings, with an explained variance of 69.0% for the first two components.

### 3D PCA

Figure S1-3: When considering the three first components in Figure Figure S1-2, we extend the accumulated explained variance to 84.3% (BCM - Baylor College of Medicine, BI - Broad Institute, BGI and WUGC - Washington University Genome Center).
