# Supplementary material for: Methodological differences can affect sequencing depth with a possible impact on the accuracy of genetic diagnosis
Source: Genet Mol Biol. 2020 Apr 27;43(2):e20190270. doi: 10.1590/1678-4685-GMB-2019-0270 (PMC7198014; doi:10.1590/1678-4685-GMB-2019-0270)
Supplement: File S4. [file 1415-4757-GMB-43-2-e20190270-s4.zip › gmb-2019-0270_20200217_suppl4.html]

Methodological differences can affect sequencing depth with possible impact on the accuracy of genetic diagnosis


# Methodological differences can affect sequencing depth with possible impact on the accuracy of genetic diagnosis

#### *Murilo G Borges, Cristiane S Rocha, Benilton S Carvalho and Iscia Lopes-Cendes*

# Supplementary file 4

## Depth variation across sequencing centers and coding impact

Figure S2-1: Heatmap showing depth variation across sequencing centers of the 450 variants with higher variance across samples. Each row represents a sample from one of the sequencing centers (BCM - Baylor College of Medicine, BI - Broad Institute, BGI and WUGC - Washington University Genome Center). 96,9% of samples (1078 of 1112) are corectly assigned to their sequencing centers when we consider 5 clusters to the dendrogram branches (right dendrogram colors). The columns represent each one of the variants, with their impact classified as high, moderate, low or modifier. We transformed data to logarithm of depth plus one (side color legend).
